# In-Silico Conceptualisation of Continuous Millifluidic Separators for Magnetic Nanoparticles

**DOI:** 10.3390/ma14216635

**Published:** 2021-11-04

**Authors:** Yanzhe Wen, Dai Jiang, Asterios Gavriilidis, Maximilian O. Besenhard

**Affiliations:** 1 Department of Chemical Engineering, University College London, Torrington Place, London WC1E 7JE, UK; yanzhe.wen.17@ucl.ac.uk; 2 Department of Electronic and Electrical Engineering, University College London, Torrington Place, London WC1E 7JE, UK; d.jiang@ucl.ac.uk; 3 School of Chemical and Process Engineering, University of Leeds, Leeds LS2 9JT, UK

**Keywords:** magnetic separation, continuous separation, magnetic nanoparticles, Lagrangian particle tracking, design optimisation, millifluidics

## Abstract

Magnetic nanoparticles are researched intensively not only for biomedical applications, but also for industrial applications including wastewater treatment and catalytic processes. Although these particles have been shown to have interesting surface properties in their bare form, their magnetisation remains a key feature, as it allows for magnetic separation. This makes them a promising carrier for precious materials and enables recovery via magnetic fields that can be turned on and off on demand, rather than using complex (nano)filtration strategies. However, designing a magnetic separator is by no means trivial, as the magnetic field and its gradient, the separator dimensions, the particle properties (such as size and susceptibility), and the throughput must be coordinated. This is showcased here for a simple continuous electromagnetic separator design requiring no expensive materials or equipment and facilitating continuous operation. The continuous electromagnetic separator chosen was based on a current-carrying wire in the centre of a capillary, which generated a radially symmetric magnetic field that could be described using cylindrical coordinates. The electromagnetic separator design was tested in-silico using a Lagrangian particle-tracking model accounting for hydrodynamics, magnetophoresis, as well as particle diffusion. This computational approach enabled the determination of separation efficiencies for varying particle sizes, magnetic field strengths, separator geometries, and flow rates, which provided insights into the complex interplay between these design parameters. In addition, the model identified the separator design allowing for the highest separation efficiency and determined the retention potential in both single and multiple separators in series. The work demonstrated that throughputs of ~1/4 L/h could be achieved for 250–500 nm iron oxide nanoparticle solutions, using less than 10 separator units in series.

## 1. Introduction

Magnetophoresis is a well-known separation concept utilising the motion of magnetic particles relative to their non-magnetic surrounding medium in response to an inhomogeneous magnetic field [1]. This contactless handling of magnetic particles, also known as “magnetic separation”, has opened up a broad field of biomedical [2,3], but also industrial, applications utilising particle phase recovery or sorting, for example, in wastewater treatment [4,5] and catalysis [6,7]. Due to the short range of magnetic field strengths decaying with 1/distance, magnetophoresis is commonly used with milli- and microscale fluid flow [8,9]. Flow chemistry examples using ferri- or ferromagnetic particles (MPs) and nanoparticles (MNPs, ≤ 500 nm) include droplet sorting [10,11], MP shape separation [12], biosensing [13,14], mixing (using diamagnetic particles) [15,16], and most prominently, cell or pathogen separation and sorting [17,18,19,20,21]. For MPs (and MNPs) to be moved by magnetophoresis, inhomogeneous magnetic fields are required. The “ends” of the MPs’ magnetic dipoles (aligned with the magnetic field) are attracted at different strengths towards the magnetic field’s north- and south pole, which yields a net movement towards the direction of the higher field strength (equivalent to a higher magnetic flux density).

As the magnetic field gradient is pivotal, magnetophoretic separation is classified as high (HGMS) [22] or low gradient magnetic separation (LGMS) [23,24]. HGMS (>100 T m^−1^) commonly uses small (i.e., with dimensions similar to the flow channel or separator geometry) permanent magnets. Although such permanent magnets can provide high-gradient magnetic fields in their proximity, their fields propagate over relatively small distances. Hence, permanent magnets are almost exclusively used for HGMS separation in microfluidics [25,26]. Alternatively, HGMS uses fine magnetic structures, such as magnetic fibres or meshes (usually out of magnetically soft materials, i.e., with a small coercivity) being magnetised by an external magnetic field generated by permanent magnets or electromagnets [27,28]. The fine magnetic matrix de-homogenises the magnetic field such that locally high magnetic field gradients occur.

Many LGMS (< 100 T m^−1^) devices are very simple, frequently involving no more than a hand-held magnet to produce the inhomogeneous magnetic field. This simplicity is an advantage over HGMS, which can present challenges in terms of complex set-up [22], operation cost and energy requirements [29], as well as particle recovery or removal [30]. Incomplete particle removal can reduce the available surface area for particle adsorption, which decreases the performance in subsequent separation cycles. A bottleneck of HGMSs for large-scale separation, either continuous or batch, is the loading capacity (maximum volume of material accumulated). MPs or MNPs accumulate first at volumes with high field gradients (e.g., close to the surfaces of magnetically soft fibres). This reduces the separation efficiency over time and can lead to plugging if the fine structures used in many HGMS devices become overloaded with magnetic material. Therefore, LGMS can be preferred for large scale magnetophoretic separation [29]. The design rules for LGMS, however, are less clear and more theoretical and experimental work must be dedicated to realise its true potential [18].

This need for design rules includes electromagnetic LGMS. Electromagnets exhibit considerably lower magnetic field gradients (with exceptions [31]), but they allow for magnetic field tuning by setting the field strengths (from 0 to a maximum value), as well as, the direction and frequency if required. This facilitates controlled MP handling, for example, for automated sorting or isolation of particulate matter based on magnetic properties [32], compact microfluidic instruments enabling automated cell separation [33], as well as magnetic systems driving nano/micro “vehicles” [34], notably including actuating systems for nanorobotics [35,36].

This work focusses on the computational design of simple electromagnetic LGMS devices for continuous MNP separation of volumes > 10 mL. First, possible separator designs and their electromagnet arrangements are discussed before the equations governing magnetophoretic separation are described for a geometrically simple design. Then, a detailed computational approach based on Lagrangian particle tracking is presented to simulate MNPs trajectories. Finally, this model is used to investigate the interplay between the magnetic separator design parameters and to identify the optimum design that yields efficient separation even at higher flow rates.

## 2. Concept and Methodology

### 2.1. Electromagnetic Separator Designs

Electromagnets generate the magnetic field via electric currents and are mostly in the form of coiled wires, where the magnetic field of DC current-carrying solenoid resembles the field of a rod magnet. Hence, electromagnets can be used similarly for localised MNP accumulation when combined with flow reactors (or, even more notably, for applications in particle accelerators) [32].

The simplest electromagnet is a straight current-carrying wire with a known magnetic field B (strictly speaking defined as magnetic flux density) of

(1) $$B=\frac{\mu_{0}\cdot I}{2\pi R}$$

in a non-magnetic medium with a relative permeability $\mu_{r}=1$. In Equation (1) I denotes the (enclosed) current, $\mu_{0}$ the vacuum permeability, and R the radial distance to the wire centre. It should be noted that Equation (1) is valid outside and inside the current-carrying wire.

**Figure 1:**
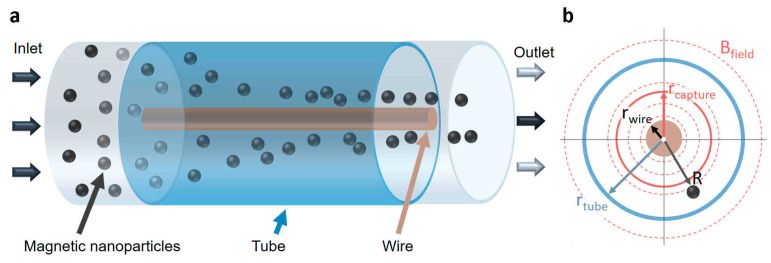
(**a**) Concept of (continuous) magnetic separator using a tube with a current-carrying wire in its centre and (**b**) front view with nomenclature.

Due to the radially symmetric magnetic field with increasing field strengths towards the wire (see Equation (1)), a magnetic separator made of tubing with a current-carrying wire in its centre, would direct MNPs towards the wire surface (see Figure 1a for the concept and Figure 1b for the nomenclature). The stronger the current, the stronger the magnetic field and the radial magnetic field gradient. The maximum current, however, needs to be capped to avoid excessive heat generation. The maximum (direct) current of a standalone wire primarily depends on the wire material used and scales with the wire cross section, i.e., with $\sim r_{wire}{}^{2}$ for (single core) wires. For example, cylindrical copper wires with radii of $r_{wire}=$0.5, 0.4 and 0.2 mm [37] allow for maximum currents of ca. 7 A, 5 A and 1 A, respectively [37]. These currents may appear high (and operation at lower currents should be aimed for) but are within the range of standard bench power supply units. In addition, the voltages required are not high due to the low wire resistivity. For example, a 500 mm long copper wire ($\rho_{Cu}=1.68\times 10^{-8}\;\Omega \;m$) with $r_{wire}=0.4$ mm requires < 0.1 V to carry 5 A resulting in a power consumption < 0.5 W. Hence, 100 W is sufficient to power 200 of such magnetic separators simultaneously.

**Figure 2:**
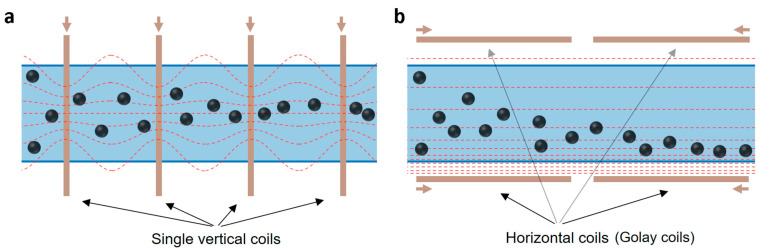
Alternative separator designs with external electromagnets (arrows point in the direction of the current) using (**a**) spaced single coils (radial gradient between single coils) and (**b**) horizontal coils in the Golay arrangement (vertical gradient). All field lines shown (dashed lines) are for illustration.

Alternatives to a current-carrying wire inside the tubing are external electromagnets, i.e., systems of current-carrying wires outside the tubing. Sufficiently spaced vertical coils are one simple design to generate a magnetic field gradient (see Figure 2a). Although there is no radial field gradient at the single coils’ axial position, a gradient forms between the coils. Computing (and optimising) the magnetic fields of single coil arrangements is not trivial, but the equations are well described [38]. The generation of field gradients via electromagnets (superconductor, but also resistive coil based) is well known for magnetic resonance imaging (MRI) [39]. Of special interest for LGMS in tubular or elongated geometries are horizontal coil arrangements used to control the vertical field gradient in MRI scanners, such as Golay coils (see Figure 2b) [40].

External electromagnets are more suitable for LGMS, as they are not limited in their dimensions and current used (the latter is limited by the heat transfer to the MNP solution), hence offer more design flexibility. Other design alternatives include periodic axial arrangements of permanent magnets alongside the tube. For example, rod magnets can be used instead of vertical coils to generate field gradients in a corresponding way to that illustrated in Figure 2a, or pairs of disc magnets can be used to generate field gradients resembling what is illustrated in Figure 2b. Furthermore, combinations of external electromagnets and soft magnetic materials can be used, such as a solenoid around the tube with a central soft magnetic wire (instead of a current carrying wire) to generate field gradients in a similar way to the design described in Figure 1. Due to the additional complexity in modelling the magnetic field of most of the designs suggested, the computational conceptualisation of a magnetic separator using a tube with a current-carrying wire in its centre is showcased in what follows. The geometric simplicity of this design allows for the use of cylindrical coordinates.

### 2.2. Modeling Magnetic Particle Transport

#### 2.2.1. Magnetophoretic Forces

For the sake of simplicity, i.e., to use cylindrical coordinates, the separator design considered is the straight current-carrying wire with a known magnetic field B (see Equation (1)). Hence, the magnetophoretic force $F_{m}$ on a magnetic particle with the volume $V_{particle}$ is given by

(2) $$F_{m}=\frac{V_{particle}\cdot \chi_{particle}}{\mu_{0}}\cdot \left(\nabla B\right)B$$

Here $\chi_{particle}$ is the dimensionless (but obtained from quantities in SI (International System) units) volumetric magnetic susceptibility. $\chi_{particle}$ was set to 3 as this is representative for magnetite (Fe_3_O_4_), i.e., the most magnetic and commonly used iron oxide phase, and the size of the iron oxide nanoparticles (IONPs) considered [41]. It should be noted, however, that using a constant for susceptibility is a simplification, especially for superparamagnetic MNPs (particles smaller ~25 nm for magnetite). In addition, Equation (1) already assumes a non-magnetic medium surrounding the particle. Due to the radial symmetry of the wire’s magnetic field (axial components are zero), the magnetic field gradient in Equation (1) is zero except in the radial direction. Hence, combining Equations (1) and (2) (using cylindrical coordinates) shows that the magnetophoresis acts only radially with a force $F_{m.radial}$ of

(3) $$F_{m.radial}=-\frac{V_{particle}\cdot I^{2}\cdot \mu_{0}\cdot \chi_{particle}}{4\pi^{2}R^{3}}$$

#### 2.2.2. Drag Forces

As magnetophoretic forces move the particles, they also experience an opposing drag force $F_{d}$. For spherical particles in laminar flow, the drag force (opposing the magnetophoresis) relates to the radial particle velocity $v_{radial}$ by Stokes’ law,

(4) $$F_{d}=6\pi \cdot r_{particle}\cdot \eta \cdot v_{radial}$$

with the particle radius $r_{particle}$ and the medium’s dynamic viscosity η. As the drag force opposing magnetophoresis increases with the particle velocity, it will increase until magnetophoretic and drag forces balance each other ($F_{m.radial}$ = $-F_{d}$). Assuming a constant field gradient, this will yield a final magnetophoretic velocity, i.e., the particles move towards the higher magnetic field density at a constant radial velocity of

(5) $$v_{radial}=\frac{F_{m,radial}}{6\pi r_{particle}\cdot \eta }=-\frac{r_{particle}{}^{2}\cdot I^{2}\cdot \mu_{0}\cdot \chi_{particle}}{18\pi^{2}\cdot \eta \cdot R^{3}}\;$$

#### 2.2.3. Particle Tracking Algorithm

**Figure 3:**
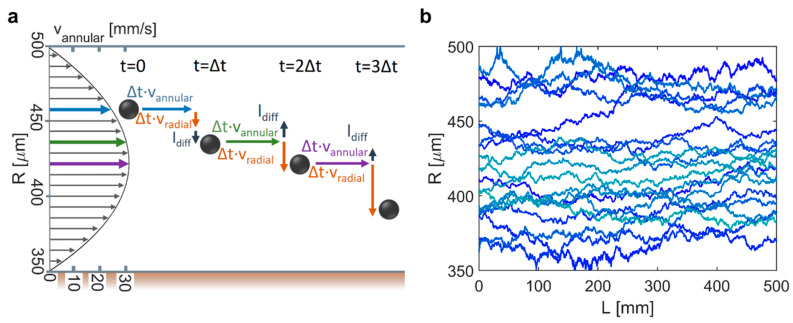
(**a**) Annular velocity profile ($\dot{V}=0.5\;\mathrm{mL}/\min$, $r_{tube}=500\;µm$, $r_{wire}=350\;µm$ ($r_{wire}$/$r_{tube}$ = 0.7) and concept of particle-tracking algorithm. (**b**) 20 trajectories of 250 nm IONPs in the magnetic separator of the same dimensions at a flow rate of 0.15 mL/min in the absence of a magnetic field.

The transport of IONPs in the magnetic separator was modelled by Lagrangian particle tracking (also known as discrete particle simulation), i.e., the individual particle trajectories were computed. Due to the small IONP sizes and the low particle concentrations considered (< 10 mg/mL), one-way coupling between the particle and liquid phase was assumed. This means that particle transport was governed by the hydrodynamics, but the hydrodynamics were not affected by the particulate phase. Furthermore, it assumes no interaction between the particles, i.e., the particle trajectories were calculated independently. In addition, gravitational forces were considered as negligible. The particle tracking algorithm used is illustrated in Figure 3 and summarised in steps I-VI below. The separator length was fixed at $L_{separator}$ =500 mm for all simulations.

**I:** The axial velocity was given by the annular Poiseuille velocity profile $v_{annular}\left(R\right)$, which was determined for the volumetric flow rate $\dot{V}$ and separator dimensions $r_{wire}$ and $r_{tube}$ by Equations (6) and (7) [42].

(6) $$v_{annular}\left(R\right)=\frac{1}{4\eta }\frac{dp}{dL}\left(r_{wire}{}^{2}-R^{2}\right)+\frac{1}{4\eta }\frac{dp}{dL}\left(r_{tube}{}^{2}-r_{wire}{}^{2}\right)\frac{\ln\left(\frac{R}{r_{wire}}\right)}{\ln\left(\frac{r_{tube}}{r_{wire}}\right)}$$

(7) $$\dot{V}=\frac{\pi }{8\eta }\frac{dp}{dL}\left(r_{tube}{}^{4}-r_{wire}{}^{4}-\frac{{\left(r_{tube}{}^{2}-r_{wire}{}^{2}\right)}^{2}}{\ln\left(\frac{r_{tube}}{r_{wire}}\right)}\right)$$

**II:** The radial position of each IONP at the separator inlet R(L = 0) was initialised randomly. The radially dependent initialisation likelihood corresponded to the axial velocity given by the annular velocity profile. This was to account for velocity-dependent particle flux into the separator when assuming that IONPs are distributed homogeneously in the solution when entering the separator.

**III:** The particle velocity in the axial direction L was governed by the particle’s radial position and the annular velocity. The axial displacement ΔL per time step Δt was updated as

(8) $$\Delta L\left(r\right)=v_{annular}\left(R\right)\cdot \Delta t$$

**IV:** The particle velocity in the radial direction R was governed by the particle’s magnetophoretic velocity $v_{radial}\left(R\right)$ given by Equation (5). The radial displacement ΔR per time step Δt was updated as

(9) $$\Delta R\left(R\right)=v_{radial}\left(R\right)\cdot \Delta t$$

**V:** Following the work of Schaller et al. [43], a stochastic Brownian motion length was added to the updated radial and axial positions to account for diffusive particle transport.

(10) $$R_{t+\Delta t}=R_{t}+\Delta R+l_{diff.\;radial}\;L_{t+\Delta t}=L_{t}+\Delta L+l_{diff.axial}$$

Here stochastic means that $l_{diff.\;radial}$ and $l_{diff.\;axial}$ were normally distributed *N*$\left(µ,\sigma^{2}\right)$ around a mean set to µ = 0 with a standard deviation $\sigma =\sqrt{2}\cdot l_{diff}$ with the diffusion length

(11) $$l_{diff}=\sqrt{D_{particle}\cdot \Delta t}$$

Note that for the sake of simplicity, Figure 3a shows only radial diffusion ($l_{diff.\;radial}$). The particle diffusion constant $D_{particle}$ in Equation (11) was estimated using the Stokes–Einstein relation

(12) $$D_{particle}=\frac{k_{B}\cdot T}{6\cdot \pi \cdot \eta \cdot r_{particle}}$$

with the Boltzmann constant $k_{B}$, the temperature T (set to 20 °C), the dynamic viscosity η (set to 1.00 mPa s, i.e., the value of water at 20 °C), and the particle radius $r_{particle}$.

In case the updated radial position exceeded the tube diameter $\left(R_{t+\Delta t}>r_{tube}\right)$, the change in radial position due to diffusion was reversed; $l_{diff.\;radial}\to -l_{diff.\;radial}$. In case the updated radial position fell below the channel width $\left(R_{t+\Delta t}+\Delta R<r_{wire}\right)$, the change in radial position was set to 0 and a counter measuring the wire collision frequency was incremented by one.

**VI:** The radial and axial particle positions were updated after each period as described in step I–V. As the particles approached the wire, the magnetophoretic forces became dominant and particles remained at the wire surface. To avoid unnecessary computational effort, particle tracking was then terminated, i.e., the particle position was not updated anymore. The tracking of particles was also terminated based on their residence time and after particles exited the separator. The three termination conditions were:

(1) The particle tracking time was a tenfold of the average residence time (referring to the liquid phase) in the separator, which was determined by the flow rate and the separator channel cross section).
(2) The updated axial position exceeded the separator length $\left(L_{t+\Delta t}>L_{separator}\right)$, i.e., the particle exited the separator.
(3) The particle collided with the wire more than 1000 times, which was determined by the collision frequency counter.

#### 2.2.4. Time-Step

Sinha et al. [44] recommended limiting the time discretisation, such that the particle movement per time step is smaller than 1/20th of the particle radius. The reasoning behind this conservative cap was to ensure that the magnetic field gradient (∇B) can be assumed as constant during Δt, such that the magnetophoretic velocity can be approximated as constant. For the maximum flow rate $v_{annular}\left(r\right)$ of the settings considered, this would require a time step < 0.001 s. As this is computationally demanding, additional, longer time intervals were tested. Therefore, identical separation conditions were simulated using different time steps (see Figure S1). These results showed that Δt=0.01 s was sufficient, hence all simulations were carried out using this time step. This larger time step was feasible due to the slower particle movement in the radial direction ($v_{radial}$) due to magnetophoresis, which allowed the assumption of a constant magnetic field gradient between time steps [43].

#### 2.2.5. Separation Efficiency Definition

After encountering one of the three termination conditions, the particle trajectory calculation finished with the particle’s final radial position $R_{final}$. The latter determined if a particle was considered as captured ($R_{final}<r_{capture}$) or not captured ($R_{final}\geq r_{capture})$. For the sake of simplicity (and for ultimate experimental implementation), $r_{capture}$ was defined as the separator channel’s geometric centre ($r_{capture}=r_{wire}+0.5\cdot \left(r_{tube}-r_{wire}\right)$). For every separation condition simulated, 10,000 particle trajectories were calculated. This large number of particle trajectories was required for comparability, as the stochastic nature of Brownian motion yielded different particle trajectories for identical separation conditions, even if particles were initialised at the same starting position R(L = 0). The capture efficiency (*CE*) reported was the percentage of particles that were classified as captured. As the annular flow profile is not symmetric around $r_{capture}$ and the radially dependent initialisation likelihood corresponded to the axial velocity (see step II in Section 2.2.3), the *CE* can be slightly above 50% for large wire-to-tube radius ratio, whilst not offering any effective separation. Therefore, the minimum required capture efficiency (*minCE*) was defined as the percentage of particles with radial positions initialised below $r_{capture}$. In the absence of magnetophoresis and assuming a radially symmetric flow profile around $r_{capture}$, the *CE* would equate to *minCE* (= 50%).

The separator efficiency (*SE*) was calculated by analogy to the vaccine efficacy [45] given by the difference in the “attack rate of disease” in unvaccinated and vaccinated individuals normalised by rate in unvaccinated individuals as

(13) $$SE=\frac{\left(100\%-minCE\right)-\left(100\%-CE\right)}{\left(100\%-minCE\right)}\times 100\%$$

In this context, an *SE* of 0% means that (on average) the particle’s radial position remained unchanged, which is expected if magnetophoresis has no effect. An *SE* of 100% means that the magnetic field was able to force all particles towards the wire and below the capture radius $\left(R_{final}<r_{capture}\right)$.

#### 2.2.6. Computation

The model used was implemented in Python 3.8.2. As each simulation to determine the capture efficiency computed 10,000 particle trajectories, the computational effort was considerable due to the interpreted nature of Python. To reduce the computation time, the Numba package in Python was used for just-in-time compilation to machine code. The Numba package reduced the computation time by more than 95% allowing, for example, determination of the separator efficiency for 5 particle radii, across 34 flowrates, using 10,000 particle trajectories (5 × 35 × 10,000 particle trajectories) in 80 min instead of 46 h using a standard computer (i7-9750H, 32.0 GB RAM; Intel, Santa Clara, CA, USA). The model results (e.g., particle trajectories and SEs) were saved as .csv files and visualised using Matlab v2020 (Mathworks, Natick, MA, USA). Files with the model used, i.e., the python code, can be downloaded from the journal’s website (see Supporting Materials).

## 3. Results

### 3.1. Effect of Design and Operating Parameters on Separator Efficiency

**Figure 4:**
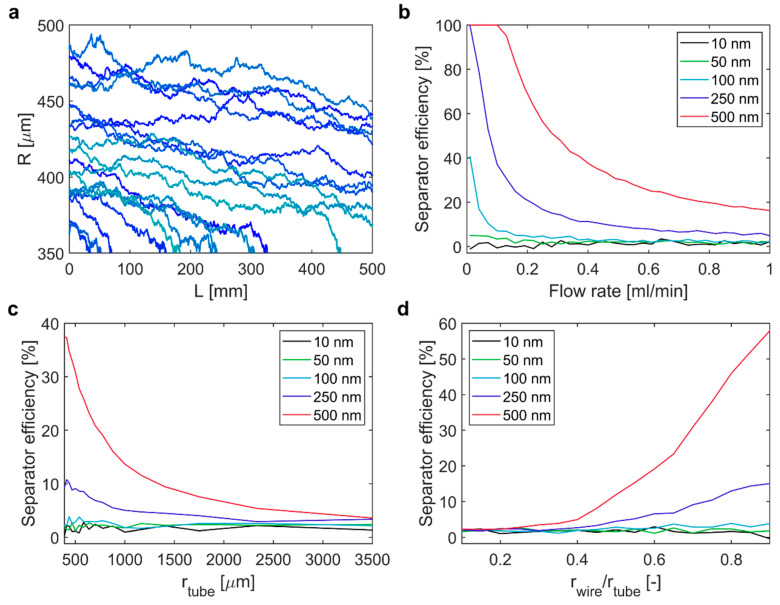
(**a**) 20 particle trajectories of 250 nm IONPs in the magnetic separator operated at 0.15 mL/min, $r_{tube}$ = 500 µm and $r_{wire}$/$r_{tube}$ = 0.7. Separator efficiency for IONP sizes as indicated at the insets for varying (**b**) flow rates ($r_{tube}$ = 500 µm, $r_{wire}$/$r_{tube}$ = 0.7), (**c**) tube radii (flow rate = 0.5 mL/min, $r_{wire}$ = 350 µm, $r_{tube}$ = 390–3500 µm, $r_{wire}$/$r_{tube}$ = 0.1–0.9) and (**d**) wire-to-tube radius ratios (flow rate = 0.5 mL/min, $r_{tube}$ = 500 µm, $r_{wire}$ = 50–450 µm, $r_{wire}$/$r_{tube}$ = 0.1–0.9).

The separator operation and design parameters affecting the efficiency are (1) the flow rate, (2) the tube radius, and (3) the wire radius (or the wire-to-tube radius ratio). The separator length was fixed ($L_{separator}$ =500 mm) and the current was determined by the wire diameter as described above. The effect of these three design parameters on the SE was tested via a sensitivity study. Therefore, each design parameter was varied separately, i.e., leaving the other parameters constant at their base case value: flow rate = 0.15 mL/min, $r_{tube}$ = 500 µm and $r_{wire}$ = 350 µm (i.e., $r_{wire}$/$r_{tube}$= 0.7). To test the SE for different particle sizes, 5 different particle radii ($r_{particle}$) between 10 and 500 nm, were considered. Figure 4a shows the particle trajectories (20 out of 10,000) of 250 nm particles in the separator operated at the base case settings. Additional particle trajectories at different flow rates were computed with and without a magnetic field (see Figure S2). The trajectories revealed the challenge of magnetophoretic separation in structures exceeding the micrometre scale. MNPs entering the separator close to the wire surface at 350 µm were quickly drawn to the wire, whereas MNPs entering close to the tubing wall at 500 µm experienced a much weaker magnetophoretic force (see Equation (3)) and converged more slowly towards the wire. This is apparent also from Figure 4b showing that lower flow rates were to the benefit of the SE. The longer residence times at lower flow rates gave particles more time to converge towards the wire. SEs of 100% were achieved at low flow rates for the otherwise constant (at their base case) design parameters for 250 nm (≤0.01 mL/min) and 500 nm (≤0.1 mL/min) particles. The 100 nm particles still showed an SE above 40% at flow rates ≤ 0.1 mL/min, but smaller particles remained almost unaffected by magnetophoresis, even at such low flow rates with SEs fluctuating close to 0%. These fluctuations originated from the stochastic nature of the diffusion as implemented in the particle tracking despite averaging over 10,000 particle trajectories.

Varying the tube radius (see Figure 4c) showed that smaller radii yield better SEs. As the flow rate was kept unchanged, the residence time decreased with the tubing radius, which was expected to result in SE decreasing, as was observed for the flow rate variations (see Figure 4b); however, the opposite trend was observed. This was due to the stronger radial dependence of the magnetophoretic force scaling with ~R^−3^ (see Equation (3)) compared to the mean residence time which scales with the separator cross section with ~R^−2^ (see Equations (6) and (7)). Hence, a closer proximity to the wire can be more beneficial to draw MNPs to the wire than a longer residence time.

The change in SE when varying the wire radius (see Figure 4d) was in line with the change in SE when varying the tube radius. A thicker wire, i.e., a larger wire-to-tube radius ratio, reduces the distance MNPs must travel, which increases the SE. In addition, a thicker wire allows for higher current, which is why the SE increased more when increasing the wire radius than when reducing the tube radius (see Figure 4c).

The sensitivity study revealed the complex interplay between the design parameters and that efficient separation of MNPs ≤ 100 nm is not readily achieved with the simple LGMS design presented. Particles larger than this (≥ 250 nm), however, can be separated efficiently with small tube radii and large wire radii, and with low flow rates. Maximising the flowrate is pivotal, as it determines the maximum separator throughput. Therefore, the separator design was optimised systematically for 250 and 500 nm MNPs for efficient separation at high flow rates.

### 3.2. Optimum Separation Conditions for 250 nm MNPs

The sensitivity study showed that varying single design parameters was helpful to identify trends, but that their complex interplay required a systematic approach to identify optimum separation conditions. Following the lessons learned from the sensitivity study, the design space for optimisation was restricted to tubing radii of 300–555 µm, and wire-to-tube radius ratios of 0.1–0.9. These restrictions also kept (1) the channel width ($r_{tube}\;$−$\;r_{wire}$) above 30 µm, and (2) the wire radius below 450 µm, so as not to exceed a current of 7 A. This optimisation design space was screened by simulating 10 tube radii, 9 wire-to-tube radius ratios, and 10 flow rates between 0.01 mL/min and 1.00 mL/min (900 simulations in total, see Table S1) computing 1000 particle trajectories each. Based on these results the search domain was narrowed using simulations computing 10,000 particle trajectories. The combination yielding the highest flow rate with SE ≥ 80% was identified as the optimum condition.

**Figure 5:**
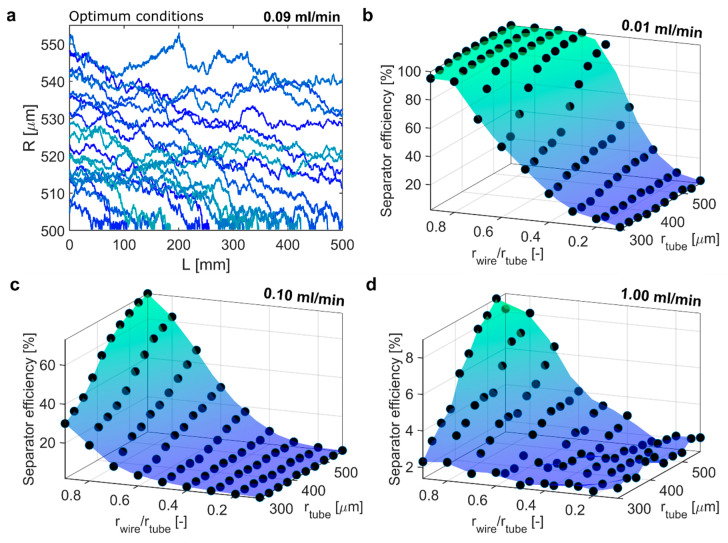
(**a**) 20 particle trajectories (out of 10,000) for the optimum separation condition for 250 nm MNPs yielding > 80% separator efficiency at 0.09 mL/min ($r_{tube}$ = 555 µm, $r_{wire}$ = 500 µm, $r_{wire}$/$r_{tube}$ = 0.9). Separator efficiency of 250 nm IONPs for different separator dimensions operated at (**b**) 0.01 mL/min, (**c**) 0.1 mL/min, and (**d**) 1 mL/min.

For 250 nm MNPs, optimum separation was achieved using a tube radius of 555 µm and a wire-to-tube radius ratio of 0.9 (i.e., a wire radius of 500 µm), which enabled an SE of 80.2% at 0.09 mL/min. Figure 5a shows the particle trajectories (20 out of 10,000) of 250 nm MNPs at these optimum separation conditions. 52% of particles touched the wire surface, highlighting the separator’s potential to retain particles efficiently.

Figure 5b–d show the SE for the design space in terms of tube and wire radii for different flow rates (0.01, 0.1 and 1 mL/min). At 0.01 mL/min an SE of 100% was achieved for wire-to-tube radius ratios larger than 0.6 and tube radii larger than 400 µm. At 0.1 mL/min none of the conditions yielded a SE higher than 74%, which was the highest SE achieved at a tube radius of 555 µm and a wire to tube radius ratio of 0.9. The latter condition (with the same separator dimensions as identified for the optimum condition) yielded the highest SE at 1 mL/min too. However, an SE lower than 10% showed that separation (using a single separator) was ineffective at this flow rate.

What was neglected for all separation conditions simulated was the temperature increase of solution due to the heat generated by the wire. We next assumed that all ohmic loss in the wire (with the resistance ${Ω}_{wire}=\rho_{Cu}\cdot L_{separator}/\left(4\pi \cdot r_{wire}{}^{2}\right)$) was transferred into the solution. The amount of heat generated is given by

(14) $$Q_{generated}=I^{2}\cdot {Ω}_{wire}$$

The resulting temperature increase of the solution passing through the separator is approximated by equating Equation (14) with the heat transferred to the solution:

(15) $$Q_{solution}=\dot{m}\cdot c_{p}\cdot \Delta T$$

Here $\dot{m}$ [g/s] is the mass flow rate of solution through the separator, $c_{p}$ [J/K] is the solution’s specific heat capacity (here water was assumed), and ΔT [K] is the temperature difference between the separator inlet and outlet. Although Equation (15) overestimates the temperature increase, as it neglects cooling at the tubing wall (or possible cooling in the wire [39]), it was used as an additional constraint, i.e., ΔT should not exceed 10 °C. As low flow rates caused the solutions to heat up, the separation efficiency had to be compromised to meet the new constraint. The same iterative approach as described above was used to find the highest SE with the additional temperature constraint. The simulations identified a best design with a tube radius of 527 µm and a wire-to-tube radius ratio of 0.9 (i.e., a wire radius of 474 µm), which enabled an SE of 13% at 0.7 mL/min. The particle trajectories plot for these conditions is shown in Figure S3a. As expected, (a higher $\dot{m\;}$ yields a lower ΔT, see Equation (15)), the higher flow rate needed to meet the temperature constraint did not allow for a high SE. In addition, only 8% of particles touched the wire, showing that multiple separators in series would be required to retain particles efficiently. The quantification of the potential to retain particles via multiple separation steps is described in the Supplementary Information (Section S3). Despite the need for multiple separators, the throughput remained comparable, as the flow rate was ~8 times higher than for the optimum separation condition not considering the temperature constraint (i.e., 0.7 mL/min instead of 0.09 mL/min). Figure S4 shows that ~9 separation steps were required to achieve the same percentage of particles touching the wire surface (MNPs retained) that was achieved for the optimum condition (52%).

### 3.3. Optimum Separation Conditions for 500 nm MNPs

**Figure 6:**
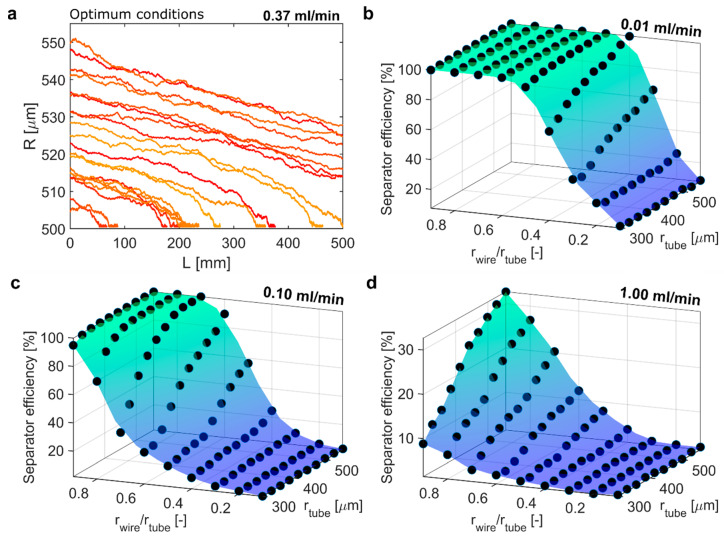
(**a**) 20 particle trajectories (out of 10,000) for the optimum separation condition for 500 nm MNPs yielding >80% separator efficiency at 0.37 mL/min ($r_{tube}$ = 555.6 µm, $r_{wire}$ = 500 µm, $r_{wire}$/$r_{tube}$ = 0.9). Separator efficiency of 500 nm IONPs for different separator dimensions operated at (**b**) 0.01 mL/min, (**c**) 0.1 mL/min, and (**d**) 1 mL/min.

To find the optimum separation conditions for 500 nm MNPs, the same procedure was applied for the same design space (see Table S2 for the initial screening). Optimum separation was achieved at a tube radius of 555 µm, a wire-to-tube ratio of 0.9, which allowed for a SE of 80.5% at 0.37 mL/min. Figure 6a shows the particle trajectories (20 out of 10,000) of 500 nm particles at these optimum separation conditions. At these optimum conditions for 500 nm MNPs, 47% of particles touched the wire surface, demonstrating the separator’s potential to retain particles efficiently at this flow rate. Figure 6b–d show the SEs of 500 nm MNPs for the optimisation design space in terms of tube and wire radii at different flow rates (0.01, 0.1 and 1 mL/min). At 0.01 mL/min, an SE of 100% was achieved for wire-to-tube radius ratios ranging between 0.6 and 0.9 and all tube radii simulated. In addition, at 0.1 mL/min, an SE of 100% could be achieved for wire-to-tube radius ratios ≥ 0.8 and tube radii larger 350 µm. At 1 mL/min none of these settings yielded an SE higher than 80% and the highest value, i.e., 32%, was achieved with the same separator dimensions as identified for the optimum conditions.

Repeating the iterative search to find the highest SE for 500 nm MNPs, considering the temperature constraint, yielded a tube radius of 527 µm and a wire-to-tube radius ratio of 0.9 (i.e., a wire radius of 474 µm) enabling an SE of 44% at 0.7 mL/min, i.e., the same separator dimensions as obtained for 250 nm MNPs considering the temperature constraint. Again, the higher flow rate (i.e., 0.7 mL/min instead of 0.37 mL/min) needed to meet the temperature requirement did not allow for an SE > 80%. Still, 26% of particles touched the wire (MNPs retained), which shows that the potential to retain particles remained even with high throughputs. As shown in Figure S4 and explained in the Supplementary Information (Section S3), 2–3 separators in series could retain particles as efficiently as the separator operated at the optimum conditions not considering the temperature constraint (see Figure S4). This is because the throughput was not reduced by much as the flow rates were ~2 times higher.

The comparison of the SEs for 250 and 500 nm particles (neglecting the temperature effect) showed that a doubling in particle size allowed for a quadrupling of the maximum flow rate (0.37 mL/min/0.09 mL/min = 4.1), which confirmed the importance of MNP size for the separator design. It should be emphasised that MNP size increase due to agglomeration or aggregation will only yield higher SEs. Hence, the simulations provide a conservative estimate of efficiencies as, due to the assumption of low particle concentrations, they did not account for (1) cooperative magnetophoresis, i.e., cooperative motion of strongly interacting MP, and (2) magnetophoresis-induced convection, i.e., the convective motion of particles towards the source of the field gradient induced by the mechanic instabilities originating from initial MNP accumulation [46]. Both effects are more likely for higher particle concentrations and larger MP sizes. However, magnetophoresis-induced convection was shown to occur for MNPs < 60 nm in the absence of cooperative magnetophoresis [47].

## 4. Conclusions and Perspective

Although low gradient magnetic separation (LGMS) devices allow for simple separator designs, their weak magnetophoretic forces require long time periods or large spatial separation domains. Therefore, LGMS devices must be designed carefully for efficient magnetic nanoparticle (MNP) separation of the process volumes required. This was demonstrated for a simple coaxial separator design using a centred current-carrying wire for magnetic field generation. This radially symmetric design (allowing the use of cylindrical coordinates) was studied computationally via a Lagrangian particle transport model accounting for convective, diffusive and magnetophoretic forces to explore the design space in terms of particle size, flow rate, as well as the tube and wire radii.

The simulations revealed that for iron oxide nanoparticle (i.e., the MNPs considered) solutions, the coaxial separator design did not yield a satisfactory separation efficiency (SE) for MNP radii ≤ 100 nm (in the absence of collective effects), but worked well for larger particles. The fact that larger MNPs could be separated more easily was expected. Choosing the optimum separation conditions, however, is not straightforward as several design parameters have opposing effects on the SE. For this reason, separator optimisation benefited from the computational strategy, which allowed the systematic screening of the design space, seeking efficient separation at high flow rates, i.e., with high throughputs.

For 250 nm MNPs, an SE > 80% was achievable for flow rates ≤ 0.09 mL/min, whereas 500 nm MNPs allowed for the same SE at 0.37 mL/min. These flow rates were attained for a separator length of 500 mm and a current capped at 7 A. Hence, higher flow rates were feasible for larger MNPs. These SEs were obtained neglecting the effect of heat generated by the wire. Repeating the simulations considering limits for the temperature increase of the solution during separation resulted in significantly lower SEs. The higher flow rates imposed by the temperature constraint, however, still facilitated efficient separation at high throughputs using multiple separators in series (instead of parallel). Nevertheless, separators using external electromagnets might be better suited for continuous LGMS in tubular separators, as they allow for more design flexibility.

The challenge the designs presented share with other LGMS devices was apparent from the simulated particle trajectories. MNPs entering the separator close to the tube wall, i.e., distant from the highest field gradients, experienced a weak magnetophoretic force and converged slowly towards the wire. This indicates that changing the separator’s channel design offers another (still unexplored) option to improve the SE. Therefore, geometries such as static mixers, pinched tubing, or other ways to temporarily narrow the laminar flow profile (especially toward the separator outlet) can further increase the SE. Such flow restrictions would allow the magnetophoretic forces to, at least temporarily, attract the MNPs and deplete the zones with weak magnetophoretic forces. Including the channel geometry as a design parameter is not straightforward and needs to take account of pressure drop, channel widths, flow rates, and the distribution of such restrictions, highlighting the importance of computational approaches to design magnetic separators.

In-silico approaches are indispensable to optimise LGMS, as finding optimum separation parameters resembles the search for a “needle in a haystack”. However, challenges remain, due to inaccurate descriptions of the spatio-temporal magnetic field strengths and the magnetic susceptibility (which depends also on the particle structure), and most importantly, the effect of cooperative magnetophoresis and magnetophoresis-induced convection for high magnetic particle concentrations.

## Data Availability

The code developed for modelling the separator is available for download (see Supporting Materials). This allows to replicate all simulations shown in this work.

## Supplementary Materials

The following are available online at https://www.mdpi.com/article/10.3390/ma14216635/s1, (1.) Supplementary Information with the sections S1: Time Discretisation, S2: Particle Trajectories (showing additional results), S3: Potential to Retain Particles via Multiple Separation Steps, S4: Initial Screening Optimisation Study 250 nm IONPs (showing raw data), S5: Initial Screening Optimisation Study 550 nm IONPs (showing raw data). (2.) The model files, i.e., the python code (In-silico_Conceptualisation_of_Continuous_Millifluidic_Separators_for_Magnetic_Nanoparticles_ModelFiles.zip).
