# Supplementary material for: In-Silico Conceptualisation of Continuous Millifluidic Separators for Magnetic Nanoparticles
Source: Materials (Basel). 2021 Nov 4;14(21):6635. doi: 10.3390/ma14216635 (PMC8586940; doi:10.3390/ma14216635)
Supplement: Supplementary file 1 [file materials-14-06635-s001.zip › materials-1352961supplementary.pdf]

materials

In-silico Conceptualisation of Continuous Millifluidic Separators for Magnetic Nanoparticles

Yanzhe Wen [1], Dai Jiang [2], Asterios Gavriilidis [1] * and Maximilian O. Besenhard [1,3] *

1. Department of Chemical Engineering, University College London, Torrington Place, London, WC1E 7JE, UK
2. Department of Electronic and Electrical Engineering, University College London, Torrington Place, London, WC1E 7JE, UK
3. School of Chemical and Process Engineering, University of Leeds, Leeds, LS2 9JT, UK

* Correspondence: a.gavriilidis@ucl.ac.uk and m.besenhard@ucl.ac.uk;

Supplementary Information

Table of Content

S1. Time Discretisation 2

S2. Particle Trajectories 2

S3. Potential to Retain Particles via Multiple Separation Steps 3

S4. Initial Screening Optimisation Study 250 nm IONPs 4

S5. Initial Screening Optimisation Study 500 nm IONPs 26

S1. Time Discretisation

In order to determine the best compromise between accuracy and computational effort three different time steps, i.e., $\Delta t = 0.1\ s$, $\Delta t = 0.01\ s$, and $\Delta t = 0.001\ s$, were used for otherwise identical simulations conditions. There was no considerable difference in the separator efficiency for all separation conditions simulated using different time steps (see Error! Reference source not found.). Therefore, $\Delta t = 0.01\ s$ was considered as sufficiently small and used for all simulations.

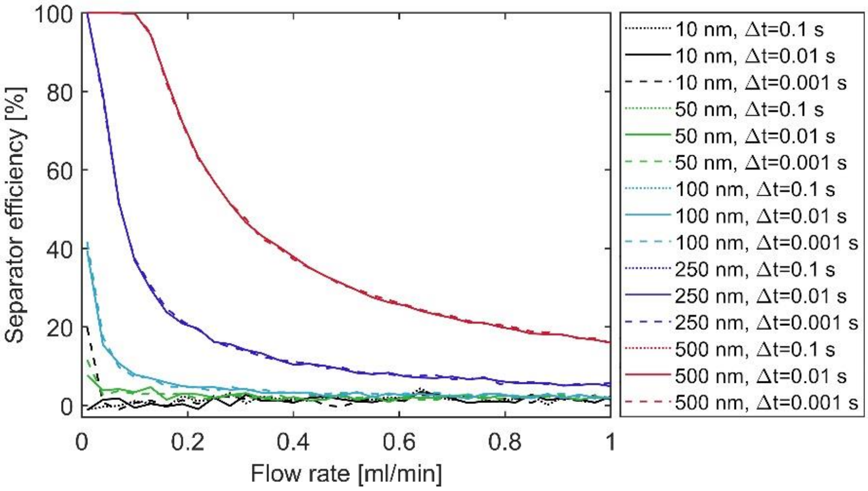

Figure S1. Reproduction of results shown in Figure 4b using different time steps (Δt= 0.1 s, 0.01 s, and 0.001 s)

S2. Particle Trajectories

Particle trajectories (20 out of 10,000 trajectories calculated) for additional separation conditions presented in **Figure 4a** are shown in **Error! Reference source not found.**.

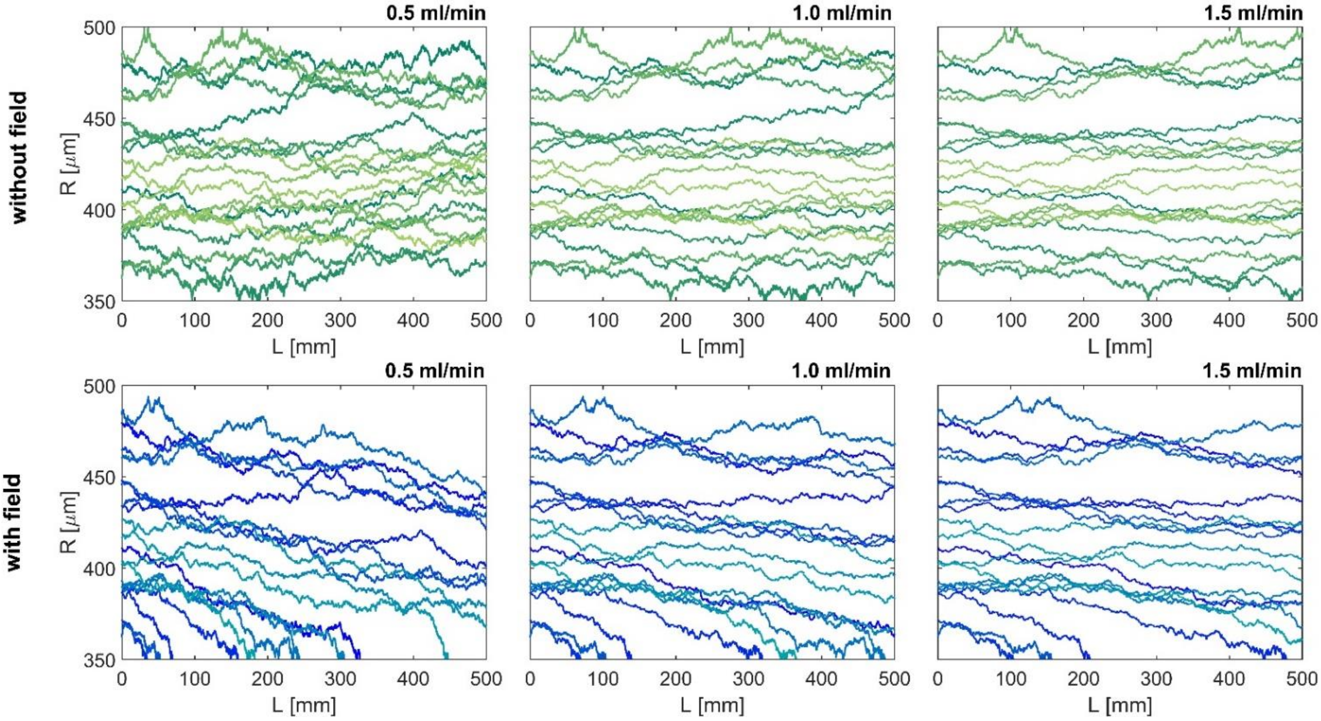

Figure S2. 20 particle trajectories of 250 nm IONPs in the magnetic separator (r_{tube}= 500 µm,and r_{wire}/r_{tube}= 0.7) operated without and with magnetic at a flowrate of 0.5 ml/min (identical to Figure 4a), 1.0 ml/min, and 1.5 ml/min.

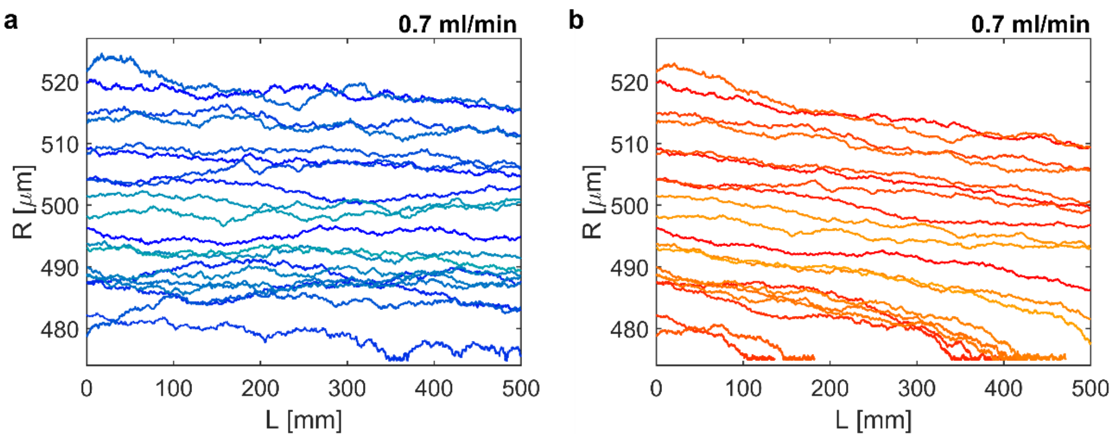

Figure S3. 20 particle trajectories (out of 10,000) for the optimum separation condition cosidering the temperature constraint ($\Delta T < 10\ °C$) for (**a**) 250 nm MNPs yielding 13% separator efficiency at 0.7 ml/min (r_{tube} = 527 µm, r_{wire} = 474 µm, r_{wire}/r_{tube} = 0.9), and (**b**) 500 nm MNPs yielding 44% separator efficiency at 0.7 ml/min (r_{tube} = 527 µm, r_{wire} = 474 µm, r_{wire}/r_{tube} = 0.9).

S3. Potential to Retain Particles via Multiple Separation Steps

The potential to retain particles was discussed in terms of the probability for particles to touch the wire in a single separation step, i.e., the percentage of MNPs that touched the wire surface when the solution passed through the separator (q_{retain}). The article reports these percentages for the optimum separation conditions for 250 and 500 nm particles, as well as the best separation conditions considering the temperature constraint for 250 and 500 nm particles.

When multiple separators are used in series, more particles can be retained. The concentration of MNPs left in solution (MNP_N) after each separation step (N) is given by

$$MNP_N = MNP_{N-1} \cdot (1 - q_{retain}/100) \quad \text{(S1)}$$

It should be highlighted that eq. S1 assusmes that the MNPs solution is homogenised before each separation step, i.e., q_{retain} is the same for each separation step as the MNPs have the same radially dependent initialisation likelihood as when entering the previous separator. This reflects the experimental constraints, since the MNP solutions would be mixed when transferred between separators. Although the structure of eq. S1 is similar to the exponential decay law (see eq. S2), the discrete nature of N requires the use of eq. S1.

$$MNP_N = MNP_{N=0} \cdot \exp(-q_{retain} \cdot N/100) \quad \text{(S2)}$$

The drop of non retained MNPs with the increase in separation steps is shown in **Error! Reference source not found.** for the four cases reported in the main article, i.e., 250 nm MNPs for optimum conditions (q_{retain} = 51.57%), 500 nm MNPs for optimum conditions (q_{retain} = 47.34%), 250 nm MNPs for conditions considering the temperature constraint (q_{retain} = 7.56%) and 500 nm MNPs for conditions considering the temperature constraint (q_{retain} = 25.46%).

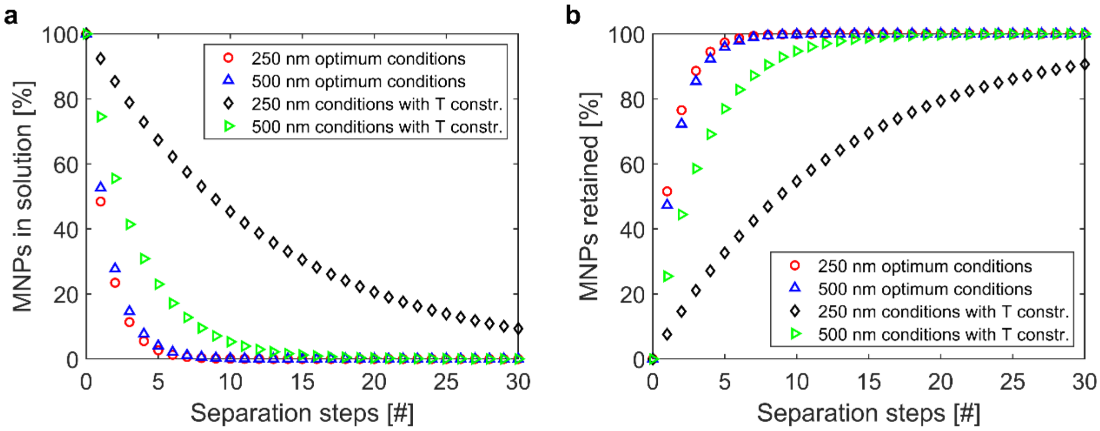

Figure S4. Relative concentration of MNPs in (**a**) solution and (**b**) retained (=100 - MNPs in solution), after multiple separation steps calculated via eq. S1 using the percentages of particles that touched the wire in a single separation step for the four cases discussed in the article; 250 nm MNPs for optimum conditions (q_{retain} = 51.57%), 500 nm MNPs for optimum conditions (q_{retain} = 47.34%), 250 nm MNPs for conditions considering the temperature constraint (q_{retain} = 7.56%), 500 nm MNPs for conditions considering the temperature constraint (q_{retain} = 25.46%).

S4. Initial Screening Optimisation Study for 250 nm IONPs

The results of the initial optimisation design space screening for 250 nm IONPs computing 1,000 particle trajectories are shown in **Table S1**.

Table S1. Capture and separator efficiency for the 900 simulations (10 tubing radii, 9 wire-to-tube radius ratios, and 10 flowrates between 0.01 ml/min and 1.00 ml/min) performed for the initial screening of the design space optimisation.

	Flowrate	Tubing radius	Wire-to-tubing rad.	Capture Efficiency	Separator Efficiency
	[ml/min]	[µm]	[-]	[%]	[%]
**1**	0.01	300	0.1	57.7	1.65
**2**	0.01	300	0.2	60.8	12.92
**3**	0.01	300	0.3	59	11.35
**4**	0.01	300	0.4	63.6	22.78
**5**	0.01	300	0.5	69.4	36.03
**6**	0.01	300	0.6	77.1	52.69
**7**	0.01	300	0.7	84.7	68.70
**8**	0.01	300	0.8	97.9	95.74
**9**	0.01	300	0.9	96.9	93.76
**10**	0.01	328.40	0.1	59.3	5.37
**11**	0.01	328.40	0.2	57.7	6.03
**12**	0.01	328.40	0.3	60.9	15.46
**13**	0.01	328.40	0.4	63.4	22.36
**14**	0.01	328.40	0.5	71.9	41.26
**15**	0.01	328.40	0.6	79.1	56.82
**16**	0.01	328.40	0.7	94.1	87.93
**17**	0.01	328.40	0.8	99.5	98.99
**18**	0.01	328.40	0.9	98.6	97.18
**19**	0.01	356.79	0.1	58.8	4.21
**20**	0.01	356.79	0.2	57	4.47
**21**	0.01	356.79	0.3	60.3	14.16
**22**	0.01	356.79	0.4	66.3	28.51
**23**	0.01	356.79	0.5	73.1	43.76
**24**	0.01	356.79	0.6	86	71.08
**25**	0.01	356.79	0.7	99.2	98.36
**26**	0.01	356.79	0.8	99.7	99.39
**27**	0.01	356.79	0.9	99.5	98.99

**28**	0.01	385.19	0.1	59.6	6.07
**29**	0.01	385.19	0.2	60.9	13.14
**30**	0.01	385.19	0.3	61.7	17.19
**31**	0.01	385.19	0.4	69.7	35.72
**32**	0.01	385.19	0.5	74.5	46.69
**33**	0.01	385.19	0.6	93	85.54
**34**	0.01	385.19	0.7	99.6	99.18
**35**	0.01	385.19	0.8	100	100.00
**36**	0.01	385.19	0.9	99.9	99.80
**37**	0.01	413.58	0.1	57.9	2.12
**38**	0.01	413.58	0.2	59.6	10.25
**39**	0.01	413.58	0.3	64.1	22.38
**40**	0.01	413.58	0.4	69.8	35.94
**41**	0.01	413.58	0.5	76	49.83
**42**	0.01	413.58	0.6	96.7	93.18
**43**	0.01	413.58	0.7	100	100.00
**44**	0.01	413.58	0.8	100	100.00
**45**	0.01	413.58	0.9	100	100.00
**46**	0.01	441.98	0.1	58.1	2.58
**47**	0.01	441.98	0.2	58.9	8.70
**48**	0.01	441.98	0.3	61.5	16.76
**49**	0.01	441.98	0.4	70.7	37.84
**50**	0.01	441.98	0.5	83	64.46
**51**	0.01	441.98	0.6	98.3	96.49
**52**	0.01	441.98	0.7	100	100.00
**53**	0.01	441.98	0.8	100	100.00
**54**	0.01	441.98	0.9	100	100.00
**55**	0.01	470.37	0.1	59.1	4.91
**56**	0.01	470.37	0.2	58.8	8.47
**57**	0.01	470.37	0.3	63.8	21.73
**58**	0.01	470.37	0.4	69.6	35.51
**59**	0.01	470.37	0.5	89.1	77.21
**60**	0.01	470.37	0.6	99	97.93
**61**	0.01	470.37	0.7	99.9	99.80
**62**	0.01	470.37	0.8	100	100.00
**63**	0.01	470.37	0.9	100	100.00
**64**	0.01	498.77	0.1	58	2.35
**65**	0.01	498.77	0.2	60.7	12.69
**66**	0.01	498.77	0.3	63.4	20.86
**67**	0.01	498.77	0.4	68.9	34.03
**68**	0.01	498.77	0.5	93.9	87.25
**69**	0.01	498.77	0.6	99.8	99.59
**70**	0.01	498.77	0.7	100	100.00
**71**	0.01	498.77	0.8	100	100.00
**72**	0.01	498.77	0.9	100	100.00
**73**	0.01	527.16	0.1	58.9	4.44
**74**	0.01	527.16	0.2	58.2	7.14
**75**	0.01	527.16	0.3	62.4	18.70
**76**	0.01	527.16	0.4	72	40.60
**77**	0.01	527.16	0.5	94.9	89.34
**78**	0.01	527.16	0.6	99.7	99.38
**79**	0.01	527.16	0.7	100	100.00
**80**	0.01	527.16	0.8	100	100.00
**81**	0.01	527.16	0.9	100	100.00

**82**	0.01	555.56	0.1	59	4.68
**83**	0.01	555.56	0.2	59.4	9.81
**84**	0.01	555.56	0.3	63.6	21.30
**85**	0.01	555.56	0.4	75.9	48.88
**86**	0.01	555.56	0.5	96.9	93.52
**87**	0.01	555.56	0.6	100	100.00
**88**	0.01	555.56	0.7	100	100.00
**89**	0.01	555.56	0.8	100	100.00
**90**	0.01	555.56	0.9	100	100.00
**91**	0.12	300	0.1	57.4	0.96
**92**	0.12	300	0.2	56.2	2.70
**93**	0.12	300	0.3	55.2	3.13
**94**	0.12	300	0.4	53.3	0.93
**95**	0.12	300	0.5	52.6	0.91
**96**	0.12	300	0.6	55.8	8.69
**97**	0.12	300	0.7	54.5	6.93
**98**	0.12	300	0.8	59.4	17.65
**99**	0.12	300	0.9	61.2	21.89
**100**	0.12	328.40	0.1	57.7	1.65
**101**	0.12	328.40	0.2	56.2	2.70
**102**	0.12	328.40	0.3	54.8	2.27
**103**	0.12	328.40	0.4	54.9	4.33
**104**	0.12	328.40	0.5	53.5	2.79
**105**	0.12	328.40	0.6	54.1	5.17
**106**	0.12	328.40	0.7	57.4	12.86
**107**	0.12	328.40	0.8	61.7	22.32
**108**	0.12	328.40	0.9	63.3	26.11
**109**	0.12	356.79	0.1	58.7	3.98
**110**	0.12	356.79	0.2	56.4	3.14
**111**	0.12	356.79	0.3	55.8	4.43
**112**	0.12	356.79	0.4	55.4	5.39
**113**	0.12	356.79	0.5	54.6	5.09
**114**	0.12	356.79	0.6	54.8	6.62
**115**	0.12	356.79	0.7	60.5	19.20
**116**	0.12	356.79	0.8	62.4	23.74
**117**	0.12	356.79	0.9	67.3	34.17
**118**	0.12	385.19	0.1	58.1	2.58
**119**	0.12	385.19	0.2	56	2.25
**120**	0.12	385.19	0.3	54.2	0.97
**121**	0.12	385.19	0.4	54.8	4.11
**122**	0.12	385.19	0.5	54.6	5.09
**123**	0.12	385.19	0.6	57.3	11.78
**124**	0.12	385.19	0.7	60.7	19.61
**125**	0.12	385.19	0.8	64	26.98
**126**	0.12	385.19	0.9	69.2	37.99
**127**	0.12	413.58	0.1	57.8	1.89
**128**	0.12	413.58	0.2	56.4	3.14
**129**	0.12	413.58	0.3	55.3	3.35
**130**	0.12	413.58	0.4	55.3	5.18
**131**	0.12	413.58	0.5	55.1	6.14
**132**	0.12	413.58	0.6	60.1	17.57
**133**	0.12	413.58	0.7	63	24.31
**134**	0.12	413.58	0.8	67.2	33.47
**135**	0.12	413.58	0.9	74	47.66

**136**	0.12	441.98	0.1	57.2	0.49
**137**	0.12	441.98	0.2	55.8	1.81
**138**	0.12	441.98	0.3	54.1	0.75
**139**	0.12	441.98	0.4	54.8	4.11
**140**	0.12	441.98	0.5	56.3	8.64
**141**	0.12	441.98	0.6	59.9	17.16
**142**	0.12	441.98	0.7	62.3	22.88
**143**	0.12	441.98	0.8	68.6	36.31
**144**	0.12	441.98	0.9	76.2	52.08
**145**	0.12	470.37	0.1	58.2	2.82
**146**	0.12	470.37	0.2	56.6	3.59
**147**	0.12	470.37	0.3	55.4	3.57
**148**	0.12	470.37	0.4	55	4.54
**149**	0.12	470.37	0.5	57.7	11.57
**150**	0.12	470.37	0.6	62.7	22.94
**151**	0.12	470.37	0.7	65	28.40
**152**	0.12	470.37	0.8	70.4	39.96
**153**	0.12	470.37	0.9	76.3	52.29
**154**	0.12	498.77	0.1	57.8	1.89
**155**	0.12	498.77	0.2	56.5	3.36
**156**	0.12	498.77	0.3	55.1	2.92
**157**	0.12	498.77	0.4	55.4	5.39
**158**	0.12	498.77	0.5	57.4	10.94
**159**	0.12	498.77	0.6	62.2	21.91
**160**	0.12	498.77	0.7	66.4	31.27
**161**	0.12	498.77	0.8	74.8	48.89
**162**	0.12	498.77	0.9	79.8	59.33
**163**	0.12	527.16	0.1	58.7	3.98
**164**	0.12	527.16	0.2	56.6	3.59
**165**	0.12	527.16	0.3	55.3	3.35
**166**	0.12	527.16	0.4	56.1	6.87
**167**	0.12	527.16	0.5	58.3	12.82
**168**	0.12	527.16	0.6	61.9	21.29
**169**	0.12	527.16	0.7	69.4	37.40
**170**	0.12	527.16	0.8	74.2	47.67
**171**	0.12	527.16	0.9	81	61.75
**172**	0.12	555.56	0.1	57.9	2.12
**173**	0.12	555.56	0.2	56	2.25
**174**	0.12	555.56	0.3	56.3	5.51
**175**	0.12	555.56	0.4	56.4	7.51
**176**	0.12	555.56	0.5	59	14.29
**177**	0.12	555.56	0.6	63.6	24.80
**178**	0.12	555.56	0.7	71.5	41.70
**179**	0.12	555.56	0.8	76.9	53.15
**180**	0.12	555.56	0.9	81.8	63.36
**181**	0.23	300	0.1	57.2	0.49
**182**	0.23	300	0.2	55	0.03
**183**	0.23	300	0.3	54.4	1.40
**184**	0.23	300	0.4	53.9	2.21
**185**	0.23	300	0.5	53.5	2.79
**186**	0.23	300	0.6	53.3	3.52
**187**	0.23	300	0.7	52.9	3.65
**188**	0.23	300	0.8	56.1	10.96
**189**	0.23	300	0.9	57	13.43

**190**	0.23	328.40	0.1	57.5	1.19
**191**	0.23	328.40	0.2	55.1	0.25
**192**	0.23	328.40	0.3	54	0.54
**193**	0.23	328.40	0.4	53.8	1.99
**194**	0.23	328.40	0.5	53.9	3.63
**195**	0.23	328.40	0.6	54.6	6.21
**196**	0.23	328.40	0.7	55	7.95
**197**	0.23	328.40	0.8	57.2	13.19
**198**	0.23	328.40	0.9	58.5	16.45
**199**	0.23	356.79	0.1	57.4	0.96
**200**	0.23	356.79	0.2	56.1	2.48
**201**	0.23	356.79	0.3	55.1	2.92
**202**	0.23	356.79	0.4	54.5	3.48
**203**	0.23	356.79	0.5	53.8	3.42
**204**	0.23	356.79	0.6	55.1	7.24
**205**	0.23	356.79	0.7	56.1	10.20
**206**	0.23	356.79	0.8	58.5	15.83
**207**	0.23	356.79	0.9	59.9	19.27
**208**	0.23	385.19	0.1	58.4	3.28
**209**	0.23	385.19	0.2	56	2.25
**210**	0.23	385.19	0.3	54.1	0.75
**211**	0.23	385.19	0.4	54	2.42
**212**	0.23	385.19	0.5	54.6	5.09
**213**	0.23	385.19	0.6	56	9.10
**214**	0.23	385.19	0.7	57.4	12.86
**215**	0.23	385.19	0.8	59.3	17.45
**216**	0.23	385.19	0.9	61.5	22.49
**217**	0.23	413.58	0.1	57.6	1.42
**218**	0.23	413.58	0.2	55.6	1.36
**219**	0.23	413.58	0.3	54.8	2.27
**220**	0.23	413.58	0.4	54.9	4.33
**221**	0.23	413.58	0.5	53.8	3.42
**222**	0.23	413.58	0.6	55.5	8.07
**223**	0.23	413.58	0.7	58	14.08
**224**	0.23	413.58	0.8	59.1	17.04
**225**	0.23	413.58	0.9	63.1	25.71
**226**	0.23	441.98	0.1	57.4	0.96
**227**	0.23	441.98	0.2	56	2.25
**228**	0.23	441.98	0.3	55.2	3.13
**229**	0.23	441.98	0.4	54.1	2.63
**230**	0.23	441.98	0.5	54.8	5.51
**231**	0.23	441.98	0.6	57	11.16
**232**	0.23	441.98	0.7	57.9	13.88
**233**	0.23	441.98	0.8	61.9	22.72
**234**	0.23	441.98	0.9	63.4	26.31
**235**	0.23	470.37	0.1	57.5	1.19
**236**	0.23	470.37	0.2	55.8	1.81
**237**	0.23	470.37	0.3	55	2.70
**238**	0.23	470.37	0.4	54.5	3.48
**239**	0.23	470.37	0.5	54.9	5.72
**240**	0.23	470.37	0.6	56.9	10.96
**241**	0.23	470.37	0.7	58.4	14.90
**242**	0.23	470.37	0.8	63	24.95
**243**	0.23	470.37	0.9	66.8	33.16

**244**	0.23	498.77	0.1	57.6	1.42
**245**	0.23	498.77	0.2	55.9	2.03
**246**	0.23	498.77	0.3	54.4	1.40
**247**	0.23	498.77	0.4	54.4	3.27
**248**	0.23	498.77	0.5	55.3	6.55
**249**	0.23	498.77	0.6	56.8	10.75
**250**	0.23	498.77	0.7	60.6	19.40
**251**	0.23	498.77	0.8	64.3	27.59
**252**	0.23	498.77	0.9	66.6	32.76
**253**	0.23	527.16	0.1	57.8	1.89
**254**	0.23	527.16	0.2	55.8	1.81
**255**	0.23	527.16	0.3	55.3	3.35
**256**	0.23	527.16	0.4	54.4	3.27
**257**	0.23	527.16	0.5	56.6	9.27
**258**	0.23	527.16	0.6	59.5	16.33
**259**	0.23	527.16	0.7	61.5	21.24
**260**	0.23	527.16	0.8	64.9	28.81
**261**	0.23	527.16	0.9	67.9	35.37
**262**	0.23	555.56	0.1	57.5	1.19
**263**	0.23	555.56	0.2	55.8	1.81
**264**	0.23	555.56	0.3	54.9	2.48
**265**	0.23	555.56	0.4	54.4	3.27
**266**	0.23	555.56	0.5	56.2	8.43
**267**	0.23	555.56	0.6	58	13.23
**268**	0.23	555.56	0.7	62	22.27
**269**	0.23	555.56	0.8	65.6	30.23
**270**	0.23	555.56	0.9	67.2	33.97
**271**	0.34	300	0.1	57.8	1.89
**272**	0.34	300	0.2	55.9	2.03
**273**	0.34	300	0.3	54	0.54
**274**	0.34	300	0.4	54.3	3.05
**275**	0.34	300	0.5	53.5	2.79
**276**	0.34	300	0.6	52.9	2.69
**277**	0.34	300	0.7	54.9	7.74
**278**	0.34	300	0.8	53.7	6.09
**279**	0.34	300	0.9	55.8	11.01
**280**	0.34	328.40	0.1	57.4	0.96
**281**	0.34	328.40	0.2	56.1	2.48
**282**	0.34	328.40	0.3	54.7	2.05
**283**	0.34	328.40	0.4	53.8	1.99
**284**	0.34	328.40	0.5	53.6	3.00
**285**	0.34	328.40	0.6	53.5	3.93
**286**	0.34	328.40	0.7	54.2	6.31
**287**	0.34	328.40	0.8	55.3	9.34
**288**	0.34	328.40	0.9	56.7	12.83
**289**	0.34	356.79	0.1	57.7	1.65
**290**	0.34	356.79	0.2	55.6	1.36
**291**	0.34	356.79	0.3	53.9	0.32
**292**	0.34	356.79	0.4	54.3	3.05
**293**	0.34	356.79	0.5	53.2	2.16
**294**	0.34	356.79	0.6	54.3	5.59
**295**	0.34	356.79	0.7	54.8	7.54
**296**	0.34	356.79	0.8	53.9	6.50
**297**	0.34	356.79	0.9	55.8	11.01

**298**	0.34	385.19	0.1	57.4	0.96
**299**	0.34	385.19	0.2	55.5	1.14
**300**	0.34	385.19	0.3	53.8	0.11
**301**	0.34	385.19	0.4	53.8	1.99
**302**	0.34	385.19	0.5	53.4	2.58
**303**	0.34	385.19	0.6	53.8	4.55
**304**	0.34	385.19	0.7	55	7.95
**305**	0.34	385.19	0.8	56.2	11.16
**306**	0.34	385.19	0.9	59.8	19.07
**307**	0.34	413.58	0.1	58.4	3.28
**308**	0.34	413.58	0.2	55.9	2.03
**309**	0.34	413.58	0.3	54.6	1.84
**310**	0.34	413.58	0.4	54.2	2.84
**311**	0.34	413.58	0.5	54.3	4.46
**312**	0.34	413.58	0.6	54.9	6.83
**313**	0.34	413.58	0.7	55.5	8.97
**314**	0.34	413.58	0.8	56.8	12.38
**315**	0.34	413.58	0.9	59.5	18.46
**316**	0.34	441.98	0.1	57.6	1.42
**317**	0.34	441.98	0.2	55.8	1.81
**318**	0.34	441.98	0.3	54	0.54
**319**	0.34	441.98	0.4	54.7	3.90
**320**	0.34	441.98	0.5	54.2	4.25
**321**	0.34	441.98	0.6	55.6	8.27
**322**	0.34	441.98	0.7	57.4	12.86
**323**	0.34	441.98	0.8	58.1	15.01
**324**	0.34	441.98	0.9	58.7	16.85
**325**	0.34	470.37	0.1	58.2	2.82
**326**	0.34	470.37	0.2	56.4	3.14
**327**	0.34	470.37	0.3	54.6	1.84
**328**	0.34	470.37	0.4	53.9	2.21
**329**	0.34	470.37	0.5	54.6	5.09
**330**	0.34	470.37	0.6	55.2	7.45
**331**	0.34	470.37	0.7	57.3	12.65
**332**	0.34	470.37	0.8	59	16.84
**333**	0.34	470.37	0.9	61.5	22.49
**334**	0.34	498.77	0.1	57.4	0.96
**335**	0.34	498.77	0.2	55.8	1.81
**336**	0.34	498.77	0.3	54.5	1.62
**337**	0.34	498.77	0.4	53.6	1.57
**338**	0.34	498.77	0.5	55	5.93
**339**	0.34	498.77	0.6	56.2	9.51
**340**	0.34	498.77	0.7	57.1	12.24
**341**	0.34	498.77	0.8	60.4	19.68
**342**	0.34	498.77	0.9	61.2	21.89
**343**	0.34	527.16	0.1	58.2	2.82
**344**	0.34	527.16	0.2	55.3	0.70
**345**	0.34	527.16	0.3	55	2.70
**346**	0.34	527.16	0.4	54.7	3.90
**347**	0.34	527.16	0.5	55.2	6.34
**348**	0.34	527.16	0.6	55.5	8.07
**349**	0.34	527.16	0.7	58.9	15.93
**350**	0.34	527.16	0.8	60.8	20.49
**351**	0.34	527.16	0.9	62.4	24.30

**352**	0.34	555.56	0.1	58	2.35
**353**	0.34	555.56	0.2	55.3	0.70
**354**	0.34	555.56	0.3	55	2.70
**355**	0.34	555.56	0.4	54.4	3.27
**356**	0.34	555.56	0.5	54.4	4.67
**357**	0.34	555.56	0.6	56.7	10.54
**358**	0.34	555.56	0.7	59.3	16.74
**359**	0.34	555.56	0.8	61.2	21.30
**360**	0.34	555.56	0.9	63	25.51
**361**	0.45	300	0.1	58.6	3.75
**362**	0.45	300	0.2	55.1	0.25
**363**	0.45	300	0.3	53.9	0.32
**364**	0.45	300	0.4	54	2.42
**365**	0.45	300	0.5	52.9	1.54
**366**	0.45	300	0.6	52.5	1.87
**367**	0.45	300	0.7	51.6	0.99
**368**	0.45	300	0.8	53.4	5.48
**369**	0.45	300	0.9	53.6	6.59
**370**	0.45	328.40	0.1	58	2.35
**371**	0.45	328.40	0.2	56.3	2.92
**372**	0.45	328.40	0.3	54.4	1.40
**373**	0.45	328.40	0.4	54.1	2.63
**374**	0.45	328.40	0.5	54	3.84
**375**	0.45	328.40	0.6	53.4	3.73
**376**	0.45	328.40	0.7	53.4	4.68
**377**	0.45	328.40	0.8	54	6.70
**378**	0.45	328.40	0.9	53.3	5.98
**379**	0.45	356.79	0.1	58	2.35
**380**	0.45	356.79	0.2	55.5	1.14
**381**	0.45	356.79	0.3	53.9	0.32
**382**	0.45	356.79	0.4	53.9	2.21
**383**	0.45	356.79	0.5	52.3	0.28
**384**	0.45	356.79	0.6	52.7	2.28
**385**	0.45	356.79	0.7	53.2	4.27
**386**	0.45	356.79	0.8	55.3	9.34
**387**	0.45	356.79	0.9	56	11.42
**388**	0.45	385.19	0.1	57.8	1.89
**389**	0.45	385.19	0.2	54.9	-0.19
**390**	0.45	385.19	0.3	54	0.54
**391**	0.45	385.19	0.4	53.6	1.57
**392**	0.45	385.19	0.5	53.1	1.95
**393**	0.45	385.19	0.6	53.7	4.35
**394**	0.45	385.19	0.7	54.1	6.11
**395**	0.45	385.19	0.8	55.3	9.34
**396**	0.45	385.19	0.9	55.9	11.22
**397**	0.45	413.58	0.1	57.8	1.89
**398**	0.45	413.58	0.2	55.9	2.03
**399**	0.45	413.58	0.3	55	2.70
**400**	0.45	413.58	0.4	53.9	2.21
**401**	0.45	413.58	0.5	52.4	0.49
**402**	0.45	413.58	0.6	53.3	3.52
**403**	0.45	413.58	0.7	54.3	6.52
**404**	0.45	413.58	0.8	55.3	9.34
**405**	0.45	413.58	0.9	56.9	13.23

**406**	0.45	441.98	0.1	57.5	1.19
**407**	0.45	441.98	0.2	55.5	1.14
**408**	0.45	441.98	0.3	54.7	2.05
**409**	0.45	441.98	0.4	53.5	1.36
**410**	0.45	441.98	0.5	53.7	3.21
**411**	0.45	441.98	0.6	54	4.97
**412**	0.45	441.98	0.7	54.2	6.31
**413**	0.45	441.98	0.8	56.7	12.18
**414**	0.45	441.98	0.9	57.6	14.64
**415**	0.45	470.37	0.1	58	2.35
**416**	0.45	470.37	0.2	56	2.25
**417**	0.45	470.37	0.3	54.7	2.05
**418**	0.45	470.37	0.4	54.2	2.84
**419**	0.45	470.37	0.5	54	3.84
**420**	0.45	470.37	0.6	54.5	6.00
**421**	0.45	470.37	0.7	55.9	9.79
**422**	0.45	470.37	0.8	57.7	14.20
**423**	0.45	470.37	0.9	59.2	17.86
**424**	0.45	498.77	0.1	58.3	3.05
**425**	0.45	498.77	0.2	55.5	1.14
**426**	0.45	498.77	0.3	54.9	2.48
**427**	0.45	498.77	0.4	53.7	1.78
**428**	0.45	498.77	0.5	53.6	3.00
**429**	0.45	498.77	0.6	55.9	8.89
**430**	0.45	498.77	0.7	55.8	9.58
**431**	0.45	498.77	0.8	58.5	15.83
**432**	0.45	498.77	0.9	60	19.47
**433**	0.45	527.16	0.1	57.6	1.42
**434**	0.45	527.16	0.2	56	2.25
**435**	0.45	527.16	0.3	54.1	0.75
**436**	0.45	527.16	0.4	53.7	1.78
**437**	0.45	527.16	0.5	54.5	4.88
**438**	0.45	527.16	0.6	55.5	8.07
**439**	0.45	527.16	0.7	56	9.99
**440**	0.45	527.16	0.8	57.9	14.61
**441**	0.45	527.16	0.9	59.8	19.07
**442**	0.45	555.56	0.1	57.8	1.89
**443**	0.45	555.56	0.2	56.1	2.48
**444**	0.45	555.56	0.3	54.3	1.19
**445**	0.45	555.56	0.4	54	2.42
**446**	0.45	555.56	0.5	54	3.84
**447**	0.45	555.56	0.6	55.5	8.07
**448**	0.45	555.56	0.7	57.8	13.68
**449**	0.45	555.56	0.8	58.5	15.83
**450**	0.45	555.56	0.9	62.2	23.90
**451**	0.56	300	0.1	57.6	1.42
**452**	0.56	300	0.2	55.6	1.36
**453**	0.56	300	0.3	54	0.54
**454**	0.56	300	0.4	54.1	2.63
**455**	0.56	300	0.5	53.3	2.37
**456**	0.56	300	0.6	53	2.90
**457**	0.56	300	0.7	53.1	4.06
**458**	0.56	300	0.8	53.7	6.09
**459**	0.56	300	0.9	53.2	5.78

**460**	0.56	328.40	0.1	57.4	0.96
**461**	0.56	328.40	0.2	55.9	2.03
**462**	0.56	328.40	0.3	54.5	1.62
**463**	0.56	328.40	0.4	53.8	1.99
**464**	0.56	328.40	0.5	52.7	1.12
**465**	0.56	328.40	0.6	53.1	3.11
**466**	0.56	328.40	0.7	53	3.86
**467**	0.56	328.40	0.8	53.8	6.29
**468**	0.56	328.40	0.9	53.6	6.59
**469**	0.56	356.79	0.1	57.7	1.65
**470**	0.56	356.79	0.2	55.8	1.81
**471**	0.56	356.79	0.3	54.5	1.62
**472**	0.56	356.79	0.4	53.5	1.36
**473**	0.56	356.79	0.5	53.7	3.21
**474**	0.56	356.79	0.6	53	2.90
**475**	0.56	356.79	0.7	53	3.86
**476**	0.56	356.79	0.8	52.7	4.06
**477**	0.56	356.79	0.9	53.9	7.19
**478**	0.56	385.19	0.1	57.9	2.12
**479**	0.56	385.19	0.2	56	2.25
**480**	0.56	385.19	0.3	54.6	1.84
**481**	0.56	385.19	0.4	54	2.42
**482**	0.56	385.19	0.5	53.5	2.79
**483**	0.56	385.19	0.6	53.5	3.93
**484**	0.56	385.19	0.7	54	5.90
**485**	0.56	385.19	0.8	54.5	7.71
**486**	0.56	385.19	0.9	55.3	10.01
**487**	0.56	413.58	0.1	57.4	0.96
**488**	0.56	413.58	0.2	55.6	1.36
**489**	0.56	413.58	0.3	54.3	1.19
**490**	0.56	413.58	0.4	53.7	1.78
**491**	0.56	413.58	0.5	53.7	3.21
**492**	0.56	413.58	0.6	54.2	5.38
**493**	0.56	413.58	0.7	53.6	5.08
**494**	0.56	413.58	0.8	54.7	8.12
**495**	0.56	413.58	0.9	55.3	10.01
**496**	0.56	441.98	0.1	57.4	0.96
**497**	0.56	441.98	0.2	56.3	2.92
**498**	0.56	441.98	0.3	54.7	2.05
**499**	0.56	441.98	0.4	54.3	3.05
**500**	0.56	441.98	0.5	54	3.84
**501**	0.56	441.98	0.6	54	4.97
**502**	0.56	441.98	0.7	54.5	6.93
**503**	0.56	441.98	0.8	55.5	9.74
**504**	0.56	441.98	0.9	57.1	13.63
**505**	0.56	470.37	0.1	57.7	1.65
**506**	0.56	470.37	0.2	55.7	1.59
**507**	0.56	470.37	0.3	55.2	3.13
**508**	0.56	470.37	0.4	54.3	3.05
**509**	0.56	470.37	0.5	53.8	3.42
**510**	0.56	470.37	0.6	54	4.97
**511**	0.56	470.37	0.7	54.5	6.93
**512**	0.56	470.37	0.8	56.6	11.97
**513**	0.56	470.37	0.9	56.2	11.82

**514**	0.56	498.77	0.1	57.7	1.65
**515**	0.56	498.77	0.2	56	2.25
**516**	0.56	498.77	0.3	54.3	1.19
**517**	0.56	498.77	0.4	53.5	1.36
**518**	0.56	498.77	0.5	53.8	3.42
**519**	0.56	498.77	0.6	54	4.97
**520**	0.56	498.77	0.7	55.2	8.36
**521**	0.56	498.77	0.8	55.9	10.55
**522**	0.56	498.77	0.9	57.3	14.03
**523**	0.56	527.16	0.1	57.7	1.65
**524**	0.56	527.16	0.2	55.9	2.03
**525**	0.56	527.16	0.3	54.7	2.05
**526**	0.56	527.16	0.4	54.1	2.63
**527**	0.56	527.16	0.5	54.3	4.46
**528**	0.56	527.16	0.6	55	7.03
**529**	0.56	527.16	0.7	55.3	8.56
**530**	0.56	527.16	0.8	56.9	12.58
**531**	0.56	527.16	0.9	57.1	13.63
**532**	0.56	555.56	0.1	57.5	1.19
**533**	0.56	555.56	0.2	55	0.03
**534**	0.56	555.56	0.3	53.9	0.32
**535**	0.56	555.56	0.4	53.8	1.99
**536**	0.56	555.56	0.5	54.1	4.04
**537**	0.56	555.56	0.6	54.3	5.59
**538**	0.56	555.56	0.7	56.6	11.22
**539**	0.56	555.56	0.8	57	12.78
**540**	0.56	555.56	0.9	57.2	13.83
**541**	0.67	300	0.1	57.6	1.42
**542**	0.67	300	0.2	55.9	2.03
**543**	0.67	300	0.3	53.8	0.11
**544**	0.67	300	0.4	53.4	1.15
**545**	0.67	300	0.5	53	1.75
**546**	0.67	300	0.6	52	0.83
**547**	0.67	300	0.7	52.2	2.22
**548**	0.67	300	0.8	52.6	3.86
**549**	0.67	300	0.9	51.8	2.96
**550**	0.67	328.40	0.1	57.5	1.19
**551**	0.67	328.40	0.2	55.5	1.14
**552**	0.67	328.40	0.3	54.6	1.84
**553**	0.67	328.40	0.4	53.7	1.78
**554**	0.67	328.40	0.5	53.5	2.79
**555**	0.67	328.40	0.6	52.5	1.87
**556**	0.67	328.40	0.7	52.2	2.22
**557**	0.67	328.40	0.8	52.9	4.47
**558**	0.67	328.40	0.9	53.6	6.59
**559**	0.67	356.79	0.1	58.4	3.28
**560**	0.67	356.79	0.2	55.9	2.03
**561**	0.67	356.79	0.3	53.9	0.32
**562**	0.67	356.79	0.4	54.4	3.27
**563**	0.67	356.79	0.5	53.2	2.16
**564**	0.67	356.79	0.6	52	0.83
**565**	0.67	356.79	0.7	54.3	6.52
**566**	0.67	356.79	0.8	53.1	4.87
**567**	0.67	356.79	0.9	52.9	5.18

**568**	0.67	385.19	0.1	58.3	3.05
**569**	0.67	385.19	0.2	55.6	1.36
**570**	0.67	385.19	0.3	55	2.70
**571**	0.67	385.19	0.4	53.8	1.99
**572**	0.67	385.19	0.5	52.9	1.54
**573**	0.67	385.19	0.6	52.9	2.69
**574**	0.67	385.19	0.7	53	3.86
**575**	0.67	385.19	0.8	54.1	6.90
**576**	0.67	385.19	0.9	53.2	5.78
**577**	0.67	413.58	0.1	57.4	0.96
**578**	0.67	413.58	0.2	55.9	2.03
**579**	0.67	413.58	0.3	54.8	2.27
**580**	0.67	413.58	0.4	54.5	3.48
**581**	0.67	413.58	0.5	53.5	2.79
**582**	0.67	413.58	0.6	53	2.90
**583**	0.67	413.58	0.7	52.6	3.04
**584**	0.67	413.58	0.8	54.7	8.12
**585**	0.67	413.58	0.9	55	9.40
**586**	0.67	441.98	0.1	58.3	3.05
**587**	0.67	441.98	0.2	56	2.25
**588**	0.67	441.98	0.3	54.3	1.19
**589**	0.67	441.98	0.4	54	2.42
**590**	0.67	441.98	0.5	52.6	0.91
**591**	0.67	441.98	0.6	53	2.90
**592**	0.67	441.98	0.7	53.9	5.70
**593**	0.67	441.98	0.8	54.3	7.31
**594**	0.67	441.98	0.9	55.7	10.81
**595**	0.67	470.37	0.1	58.3	3.05
**596**	0.67	470.37	0.2	56.1	2.48
**597**	0.67	470.37	0.3	54.4	1.40
**598**	0.67	470.37	0.4	54	2.42
**599**	0.67	470.37	0.5	53.5	2.79
**600**	0.67	470.37	0.6	53.3	3.52
**601**	0.67	470.37	0.7	53.4	4.68
**602**	0.67	470.37	0.8	55	8.73
**603**	0.67	470.37	0.9	56.3	12.02
**604**	0.67	498.77	0.1	57.7	1.65
**605**	0.67	498.77	0.2	55.8	1.81
**606**	0.67	498.77	0.3	54.2	0.97
**607**	0.67	498.77	0.4	53.6	1.57
**608**	0.67	498.77	0.5	53.4	2.58
**609**	0.67	498.77	0.6	52.9	2.69
**610**	0.67	498.77	0.7	53.5	4.88
**611**	0.67	498.77	0.8	55	8.73
**612**	0.67	498.77	0.9	56.4	12.22
**613**	0.67	527.16	0.1	58.3	3.05
**614**	0.67	527.16	0.2	55.7	1.59
**615**	0.67	527.16	0.3	54.4	1.40
**616**	0.67	527.16	0.4	54.1	2.63
**617**	0.67	527.16	0.5	53.9	3.63
**618**	0.67	527.16	0.6	53.4	3.73
**619**	0.67	527.16	0.7	54.3	6.52
**620**	0.67	527.16	0.8	57.1	12.99
**621**	0.67	527.16	0.9	56	11.42

**622**	0.67	555.56	0.1	57.2	0.49
**623**	0.67	555.56	0.2	55	0.03
**624**	0.67	555.56	0.3	54.2	0.97
**625**	0.67	555.56	0.4	54.1	2.63
**626**	0.67	555.56	0.5	54.2	4.25
**627**	0.67	555.56	0.6	54.5	6.00
**628**	0.67	555.56	0.7	55.4	8.77
**629**	0.67	555.56	0.8	56	10.76
**630**	0.67	555.56	0.9	57.6	14.64
**631**	0.78	300	0.1	58.1	2.58
**632**	0.78	300	0.2	55.5	1.14
**633**	0.78	300	0.3	53.8	0.11
**634**	0.78	300	0.4	53.8	1.99
**635**	0.78	300	0.5	52.6	0.91
**636**	0.78	300	0.6	51.8	0.42
**637**	0.78	300	0.7	51.7	1.20
**638**	0.78	300	0.8	52.3	3.25
**639**	0.78	300	0.9	52.6	4.57
**640**	0.78	328.40	0.1	58.1	2.58
**641**	0.78	328.40	0.2	56.7	3.81
**642**	0.78	328.40	0.3	54.4	1.40
**643**	0.78	328.40	0.4	53.4	1.15
**644**	0.78	328.40	0.5	53	1.75
**645**	0.78	328.40	0.6	52.4	1.66
**646**	0.78	328.40	0.7	51.9	1.61
**647**	0.78	328.40	0.8	52.3	3.25
**648**	0.78	328.40	0.9	52.3	3.97
**649**	0.78	356.79	0.1	57.8	1.89
**650**	0.78	356.79	0.2	56	2.25
**651**	0.78	356.79	0.3	53.8	0.11
**652**	0.78	356.79	0.4	53.9	2.21
**653**	0.78	356.79	0.5	52.4	0.49
**654**	0.78	356.79	0.6	52.4	1.66
**655**	0.78	356.79	0.7	53	3.86
**656**	0.78	356.79	0.8	53.4	5.48
**657**	0.78	356.79	0.9	52.9	5.18
**658**	0.78	385.19	0.1	58.1	2.58
**659**	0.78	385.19	0.2	55.2	0.48
**660**	0.78	385.19	0.3	54.8	2.27
**661**	0.78	385.19	0.4	53.8	1.99
**662**	0.78	385.19	0.5	52.8	1.33
**663**	0.78	385.19	0.6	53.3	3.52
**664**	0.78	385.19	0.7	53.1	4.06
**665**	0.78	385.19	0.8	53.8	6.29
**666**	0.78	385.19	0.9	52.8	4.97
**667**	0.78	413.58	0.1	58.1	2.58
**668**	0.78	413.58	0.2	55.8	1.81
**669**	0.78	413.58	0.3	54.4	1.40
**670**	0.78	413.58	0.4	54.3	3.05
**671**	0.78	413.58	0.5	52.9	1.54
**672**	0.78	413.58	0.6	53.1	3.11
**673**	0.78	413.58	0.7	52.6	3.04
**674**	0.78	413.58	0.8	53.7	6.09
**675**	0.78	413.58	0.9	53.9	7.19

**676**	0.78	441.98	0.1	57.8	1.89
**677**	0.78	441.98	0.2	55.9	2.03
**678**	0.78	441.98	0.3	54.1	0.75
**679**	0.78	441.98	0.4	54.1	2.63
**680**	0.78	441.98	0.5	52.9	1.54
**681**	0.78	441.98	0.6	53.7	4.35
**682**	0.78	441.98	0.7	52.9	3.65
**683**	0.78	441.98	0.8	54.6	7.92
**684**	0.78	441.98	0.9	54.9	9.20
**685**	0.78	470.37	0.1	57.7	1.65
**686**	0.78	470.37	0.2	55.5	1.14
**687**	0.78	470.37	0.3	54.7	2.05
**688**	0.78	470.37	0.4	53.8	1.99
**689**	0.78	470.37	0.5	53.4	2.58
**690**	0.78	470.37	0.6	53.4	3.73
**691**	0.78	470.37	0.7	54.1	6.11
**692**	0.78	470.37	0.8	54.2	7.10
**693**	0.78	470.37	0.9	54.4	8.20
**694**	0.78	498.77	0.1	58.2	2.82
**695**	0.78	498.77	0.2	55.8	1.81
**696**	0.78	498.77	0.3	55.4	3.57
**697**	0.78	498.77	0.4	53.8	1.99
**698**	0.78	498.77	0.5	53	1.75
**699**	0.78	498.77	0.6	53.5	3.93
**700**	0.78	498.77	0.7	54.2	6.31
**701**	0.78	498.77	0.8	53.8	6.29
**702**	0.78	498.77	0.9	56	11.42
**703**	0.78	527.16	0.1	57.9	2.12
**704**	0.78	527.16	0.2	56	2.25
**705**	0.78	527.16	0.3	54.9	2.48
**706**	0.78	527.16	0.4	54.7	3.90
**707**	0.78	527.16	0.5	53.4	2.58
**708**	0.78	527.16	0.6	53.3	3.52
**709**	0.78	527.16	0.7	54.3	6.52
**710**	0.78	527.16	0.8	55.9	10.55
**711**	0.78	527.16	0.9	55.7	10.81
**712**	0.78	555.56	0.1	57.6	1.42
**713**	0.78	555.56	0.2	56	2.25
**714**	0.78	555.56	0.3	54	0.54
**715**	0.78	555.56	0.4	53.6	1.57
**716**	0.78	555.56	0.5	54	3.84
**717**	0.78	555.56	0.6	53.9	4.76
**718**	0.78	555.56	0.7	55.4	8.77
**719**	0.78	555.56	0.8	55.9	10.55
**720**	0.78	555.56	0.9	56.4	12.22
**721**	0.89	300	0.1	57.8	1.89
**722**	0.89	300	0.2	55.7	1.59
**723**	0.89	300	0.3	54.2	0.97
**724**	0.89	300	0.4	53.4	1.15
**725**	0.89	300	0.5	52.9	1.54
**726**	0.89	300	0.6	52.2	1.25
**727**	0.89	300	0.7	52.1	2.02
**728**	0.89	300	0.8	52.2	3.05
**729**	0.89	300	0.9	51.3	1.95

**730**	0.89	328.40	0.1	58.3	3.05
**731**	0.89	328.40	0.2	55.8	1.81
**732**	0.89	328.40	0.3	54.5	1.62
**733**	0.89	328.40	0.4	53.6	1.57
**734**	0.89	328.40	0.5	52.5	0.70
**735**	0.89	328.40	0.6	51.9	0.63
**736**	0.89	328.40	0.7	51.6	0.99
**737**	0.89	328.40	0.8	52.4	3.45
**738**	0.89	328.40	0.9	51.3	1.95
**739**	0.89	356.79	0.1	57.4	0.96
**740**	0.89	356.79	0.2	55.5	1.14
**741**	0.89	356.79	0.3	54.3	1.19
**742**	0.89	356.79	0.4	53.1	0.51
**743**	0.89	356.79	0.5	54.4	4.67
**744**	0.89	356.79	0.6	52.4	1.66
**745**	0.89	356.79	0.7	52.2	2.22
**746**	0.89	356.79	0.8	52.6	3.86
**747**	0.89	356.79	0.9	53.4	6.18
**748**	0.89	385.19	0.1	58	2.35
**749**	0.89	385.19	0.2	55.6	1.36
**750**	0.89	385.19	0.3	54.5	1.62
**751**	0.89	385.19	0.4	53.8	1.99
**752**	0.89	385.19	0.5	53	1.75
**753**	0.89	385.19	0.6	52.7	2.28
**754**	0.89	385.19	0.7	51.9	1.61
**755**	0.89	385.19	0.8	52.5	3.66
**756**	0.89	385.19	0.9	54.3	7.99
**757**	0.89	413.58	0.1	58	2.35
**758**	0.89	413.58	0.2	56.2	2.70
**759**	0.89	413.58	0.3	54.8	2.27
**760**	0.89	413.58	0.4	53.4	1.14
**761**	0.89	413.58	0.5	52.6	0.91
**762**	0.89	413.58	0.6	52.4	1.66
**763**	0.89	413.58	0.7	52.6	3.04
**764**	0.89	413.58	0.8	53.5	5.68
**765**	0.89	413.58	0.9	53.7	6.79
**766**	0.89	441.98	0.1	57.8	1.89
**767**	0.89	441.98	0.2	56.1	2.48
**768**	0.89	441.98	0.3	54.1	0.75
**769**	0.89	441.98	0.4	53.3	0.93
**770**	0.89	441.98	0.5	53.4	2.58
**771**	0.89	441.98	0.6	52.7	2.28
**772**	0.89	441.98	0.7	52.9	3.65
**773**	0.89	441.98	0.8	53.8	6.29
**774**	0.89	441.98	0.9	53.6	6.59
**775**	0.89	470.37	0.1	57.8	1.89
**776**	0.89	470.37	0.2	55.9	2.03
**777**	0.89	470.37	0.3	54.5	1.62
**778**	0.89	470.37	0.4	53.3	0.93
**779**	0.89	470.37	0.5	52.5	0.70
**780**	0.89	470.37	0.6	53.7	4.35
**781**	0.89	470.37	0.7	53.4	4.68
**782**	0.89	470.37	0.8	54.1	6.90
**783**	0.89	470.37	0.9	54.4	8.20

**784**	0.89	498.77	0.1	57.9	2.12
**785**	0.89	498.77	0.2	55.5	1.14
**786**	0.89	498.77	0.3	54.4	1.40
**787**	0.89	498.77	0.4	53.4	1.14
**788**	0.89	498.77	0.5	53.3	2.37
**789**	0.89	498.77	0.6	53.4	3.73
**790**	0.89	498.77	0.7	53.4	4.68
**791**	0.89	498.77	0.8	53.6	5.89
**792**	0.89	498.77	0.9	54.3	7.99
**793**	0.89	527.16	0.1	57.8	1.89
**794**	0.89	527.16	0.2	56.2	2.70
**795**	0.89	527.16	0.3	54.2	0.97
**796**	0.89	527.16	0.4	53.5	1.36
**797**	0.89	527.16	0.5	53.4	2.58
**798**	0.89	527.16	0.6	53.4	3.73
**799**	0.89	527.16	0.7	53.6	5.08
**800**	0.89	527.16	0.8	54.4	7.51
**801**	0.89	527.16	0.9	54.7	8.80
**802**	0.89	555.56	0.1	57.8	1.89
**803**	0.89	555.56	0.2	55.5	1.14
**804**	0.89	555.56	0.3	54.7	2.05
**805**	0.89	555.56	0.4	54	2.42
**806**	0.89	555.56	0.5	54	3.84
**807**	0.89	555.56	0.6	53.7	4.35
**808**	0.89	555.56	0.7	54.6	7.13
**809**	0.89	555.56	0.8	55.1	8.93
**810**	0.89	555.56	0.9	55.5	10.41
**811**	1	300	0.1	58.3	3.05
**812**	1	300	0.2	54.8	-0.41
**813**	1	300	0.3	54.4	1.40
**814**	1	300	0.4	53.3	0.93
**815**	1	300	0.5	52.7	1.12
**816**	1	300	0.6	52.3	1.45
**817**	1	300	0.7	52	1.81
**818**	1	300	0.8	51.4	1.43
**819**	1	300	0.9	51.6	2.56
**820**	1	328.40	0.1	57.3	0.72
**821**	1	328.40	0.2	56.1	2.48
**822**	1	328.40	0.3	54.8	2.27
**823**	1	328.40	0.4	54	2.42
**824**	1	328.40	0.5	53.1	1.95
**825**	1	328.40	0.6	51.8	0.42
**826**	1	328.40	0.7	52.2	2.22
**827**	1	328.40	0.8	51.8	2.24
**828**	1	328.40	0.9	52.2	3.77
**829**	1	356.79	0.1	58.2	2.82
**830**	1	356.79	0.2	55.3	0.70
**831**	1	356.79	0.3	54.5	1.62
**832**	1	356.79	0.4	53.7	1.78
**833**	1	356.79	0.5	53.1	1.95
**834**	1	356.79	0.6	52.5	1.87
**835**	1	356.79	0.7	52.5	2.83
**836**	1	356.79	0.8	52.1	2.85
**837**	1	356.79	0.9	52.2	3.77

**838**	1	385.19	0.1	57.6	1.42
**839**	1	385.19	0.2	55.9	2.03
**840**	1	385.19	0.3	54.1	0.75
**841**	1	385.19	0.4	53.7	1.78
**842**	1	385.19	0.5	53.3	2.37
**843**	1	385.19	0.6	52.7	2.28
**844**	1	385.19	0.7	52.2	2.22
**845**	1	385.19	0.8	52.1	2.85
**846**	1	385.19	0.9	53.4	6.18
**847**	1	413.58	0.1	57.6	1.42
**848**	1	413.58	0.2	56	2.25
**849**	1	413.58	0.3	54.4	1.40
**850**	1	413.58	0.4	53.3	0.93
**851**	1	413.58	0.5	53.2	2.16
**852**	1	413.58	0.6	53.2	3.31
**853**	1	413.58	0.7	52.7	3.24
**854**	1	413.58	0.8	53.4	5.48
**855**	1	413.58	0.9	53.4	6.18
**856**	1	441.98	0.1	57.8	1.89
**857**	1	441.98	0.2	55.7	1.59
**858**	1	441.98	0.3	54.7	2.05
**859**	1	441.98	0.4	53.9	2.21
**860**	1	441.98	0.5	53	1.75
**861**	1	441.98	0.6	52.8	2.49
**862**	1	441.98	0.7	52.9	3.65
**863**	1	441.98	0.8	53.7	6.09
**864**	1	441.98	0.9	53.5	6.38
**865**	1	470.37	0.1	57.6	1.42
**866**	1	470.37	0.2	56	2.25
**867**	1	470.37	0.3	54.6	1.84
**868**	1	470.37	0.4	53.8	1.99
**869**	1	470.37	0.5	53.1	1.95
**870**	1	470.37	0.6	53.4	3.73
**871**	1	470.37	0.7	53.3	4.47
**872**	1	470.37	0.8	53.8	6.29
**873**	1	470.37	0.9	53.7	6.79
**874**	1	498.77	0.1	57.6	1.42
**875**	1	498.77	0.2	56.2	2.70
**876**	1	498.77	0.3	54.3	1.19
**877**	1	498.77	0.4	53.1	0.51
**878**	1	498.77	0.5	52.5	0.70
**879**	1	498.77	0.6	53.6	4.14
**880**	1	498.77	0.7	52.4	2.63
**881**	1	498.77	0.8	54.3	7.31
**882**	1	498.77	0.9	53.3	5.98
**883**	1	527.16	0.1	58.1	2.58
**884**	1	527.16	0.2	55.6	1.36
**885**	1	527.16	0.3	54.5	1.62
**886**	1	527.16	0.4	53.4	1.14
**887**	1	527.16	0.5	53.8	3.42
**888**	1	527.16	0.6	53.5	3.93
**889**	1	527.16	0.7	53.2	4.27
**890**	1	527.16	0.8	53.8	6.29
**891**	1	527.16	0.9	54.3	7.99

**892**	1	555.56	0.1	57.6	1.42
**893**	1	555.56	0.2	55.7	1.59
**894**	1	555.56	0.3	54.8	2.27
**895**	1	555.56	0.4	53.9	2.21
**896**	1	555.56	0.5	54.1	4.04
**897**	1	555.56	0.6	53.5	3.93
**898**	1	555.56	0.7	53.6	5.08
**899**	1	555.56	0.8	54.2	7.10
**900**	1	555.56	0.9	54.5	8.40

S5. Initial Screening Optimisation Study 500 nm IONPs

The results of the initial optimisation design space screening for 500 nm IONPs computing 1,000 particle trajectories are shown in Table S2.

Table S2. Capture and separator efficiency for the 900 simulations (10 tubing radii, 9 wire-to-tube radius ratios, and 10 flowrates between 0.01 ml/min and 1.00 ml/min) performed for the initial screening of the design space optimisation.

	**Flowrate [ml/min]**	**Tubing radius [µm]**	**Wire-to-tubing rad. [-]**	**Capture Efficiency [%]**	**Separator Efficiency [%]**
**1**	0.01	300	0.1	60.8	8.86
**2**	0.01	300	0.2	65.8	24.02
**3**	0.01	300	0.3	71.4	38.16
**4**	0.01	300	0.4	84.3	66.69
**5**	0.01	300	0.5	97.1	93.94
**6**	0.01	300	0.6	100	100.00
**7**	0.01	300	0.7	100	100.00
**8**	0.01	300	0.8	100	100.00
**9**	0.01	300	0.9	100	100.00
**10**	0.01	328.40	0.1	61.2	9.79
**11**	0.01	328.40	0.2	63.6	19.14
**12**	0.01	328.40	0.3	70.8	36.86
**13**	0.01	328.40	0.4	87.4	73.27
**14**	0.01	328.40	0.5	99	97.91
**15**	0.01	328.40	0.6	100	100.00
**16**	0.01	328.40	0.7	100	100.00
**17**	0.01	328.40	0.8	100	100.00
**18**	0.01	328.40	0.9	100	100.00
**19**	0.01	356.79	0.1	60.7	8.63
**20**	0.01	356.79	0.2	63.5	18.91
**21**	0.01	356.79	0.3	71.6	38.59
**22**	0.01	356.79	0.4	89.2	77.09
**23**	0.01	356.79	0.5	98.6	97.07
**24**	0.01	356.79	0.6	100	100.00
**25**	0.01	356.79	0.7	100	100.00
**26**	0.01	356.79	0.8	100	100.00
**27**	0.01	356.79	0.9	100	100.00
**28**	0.01	385.19	0.1	61.5	10.49
**29**	0.01	385.19	0.2	65.5	23.36
**30**	0.01	385.19	0.3	74.1	44.00
**31**	0.01	385.19	0.4	92.4	83.88
**32**	0.01	385.19	0.5	99.5	98.95
**33**	0.01	385.19	0.6	100	100.00
**34**	0.01	385.19	0.7	100	100.00

**35**	0.01	385.19	0.8	100	100.00
**36**	0.01	385.19	0.9	100	100.00
**37**	0.01	413.58	0.1	59.6	6.07
**38**	0.01	413.58	0.2	64.8	21.80
**39**	0.01	413.58	0.3	77.1	50.49
**40**	0.01	413.58	0.4	93.5	86.21
**41**	0.01	413.58	0.5	100	100.00
**42**	0.01	413.58	0.6	100	100.00
**43**	0.01	413.58	0.7	100	100.00
**44**	0.01	413.58	0.8	100	100.00
**45**	0.01	413.58	0.9	100	100.00
**46**	0.01	441.98	0.1	60.1	7.23
**47**	0.01	441.98	0.2	64.1	20.25
**48**	0.01	441.98	0.3	77.8	52.00
**49**	0.01	441.98	0.4	95.3	90.03
**50**	0.01	441.98	0.5	100	100.00
**51**	0.01	441.98	0.6	100	100.00
**52**	0.01	441.98	0.7	100	100.00
**53**	0.01	441.98	0.8	100	100.00
**54**	0.01	441.98	0.9	100	100.00
**55**	0.01	470.37	0.1	61.9	11.42
**56**	0.01	470.37	0.2	65.6	23.58
**57**	0.01	470.37	0.3	79.9	56.54
**58**	0.01	470.37	0.4	94.8	88.97
**59**	0.01	470.37	0.5	100	100.00
**60**	0.01	470.37	0.6	100	100.00
**61**	0.01	470.37	0.7	100	100.00
**62**	0.01	470.37	0.8	100	100.00
**63**	0.01	470.37	0.9	100	100.00
**64**	0.01	498.77	0.1	61.4	10.26
**65**	0.01	498.77	0.2	65.8	24.02
**66**	0.01	498.77	0.3	81.2	59.35
**67**	0.01	498.77	0.4	96.8	93.21
**68**	0.01	498.77	0.5	100	100.00
**69**	0.01	498.77	0.6	100	100.00
**70**	0.01	498.77	0.7	100	100.00
**71**	0.01	498.77	0.8	100	100.00
**72**	0.01	498.77	0.9	100	100.00
**73**	0.01	527.16	0.1	60.7	8.63
**74**	0.01	527.16	0.2	65.8	24.02
**75**	0.01	527.16	0.3	82.4	61.95
**76**	0.01	527.16	0.4	99	97.88
**77**	0.01	527.16	0.5	100	100.00
**78**	0.01	527.16	0.6	100	100.00
**79**	0.01	527.16	0.7	100	100.00
**80**	0.01	527.16	0.8	100	100.00
**81**	0.01	527.16	0.9	100	100.00
**82**	0.01	555.56	0.1	61	9.33
**83**	0.01	555.56	0.2	65.9	24.25
**84**	0.01	555.56	0.3	82.8	62.81
**85**	0.01	555.56	0.4	99.9	99.79
**86**	0.01	555.56	0.5	100	100.00
**87**	0.01	555.56	0.6	100	100.00
**88**	0.01	555.56	0.7	100	100.00

**89**	0.01	555.56	0.8	100	100.00
**90**	0.01	555.56	0.9	100	100.00
**91**	0.12	300	0.1	57.6	1.42
**92**	0.12	300	0.2	56.6	3.59
**93**	0.12	300	0.3	56.2	5.30
**94**	0.12	300	0.4	56.4	7.51
**95**	0.12	300	0.5	57.8	11.78
**96**	0.12	300	0.6	63.3	24.18
**97**	0.12	300	0.7	66.3	31.06
**98**	0.12	300	0.8	80.3	60.04
**99**	0.12	300	0.9	93.1	86.11
**100**	0.12	328.40	0.1	58	2.35
**101**	0.12	328.40	0.2	56.5	3.36
**102**	0.12	328.40	0.3	56.6	6.16
**103**	0.12	328.40	0.4	57.1	8.99
**104**	0.12	328.40	0.5	58.7	13.66
**105**	0.12	328.40	0.6	62.7	22.94
**106**	0.12	328.40	0.7	72.1	42.93
**107**	0.12	328.40	0.8	88.6	76.88
**108**	0.12	328.40	0.9	98.2	96.38
**109**	0.12	356.79	0.1	58.3	3.05
**110**	0.12	356.79	0.2	57.1	4.70
**111**	0.12	356.79	0.3	56.6	6.16
**112**	0.12	356.79	0.4	58	10.90
**113**	0.12	356.79	0.5	60.9	18.26
**114**	0.12	356.79	0.6	65	27.69
**115**	0.12	356.79	0.7	79.5	58.07
**116**	0.12	356.79	0.8	93	85.80
**117**	0.12	356.79	0.9	98.4	96.78
**118**	0.12	385.19	0.1	58.6	3.75
**119**	0.12	385.19	0.2	56	2.25
**120**	0.12	385.19	0.3	56	4.86
**121**	0.12	385.19	0.4	59.2	13.45
**122**	0.12	385.19	0.5	60.1	16.59
**123**	0.12	385.19	0.6	71.3	40.71
**124**	0.12	385.19	0.7	84.2	67.68
**125**	0.12	385.19	0.8	96.9	93.71
**126**	0.12	385.19	0.9	100	100.00
**127**	0.12	413.58	0.1	57.7	1.65
**128**	0.12	413.58	0.2	56.8	4.03
**129**	0.12	413.58	0.3	56.2	5.30
**130**	0.12	413.58	0.4	58.5	11.96
**131**	0.12	413.58	0.5	61	18.47
**132**	0.12	413.58	0.6	74.5	47.32
**133**	0.12	413.58	0.7	87	73.41
**134**	0.12	413.58	0.8	98.8	97.57
**135**	0.12	413.58	0.9	100	100.00
**136**	0.12	441.98	0.1	57.6	1.42
**137**	0.12	441.98	0.2	56.4	3.14
**138**	0.12	441.98	0.3	56.1	5.08
**139**	0.12	441.98	0.4	58.5	11.96
**140**	0.12	441.98	0.5	65.3	27.46
**141**	0.12	441.98	0.6	78.9	56.41
**142**	0.12	441.98	0.7	92.2	84.04

**143**	0.12	441.98	0.8	99.8	99.59
**144**	0.12	441.98	0.9	100	100.00
**145**	0.12	470.37	0.1	58.4	3.28
**146**	0.12	470.37	0.2	56.7	3.81
**147**	0.12	470.37	0.3	56.6	6.16
**148**	0.12	470.37	0.4	59.2	13.45
**149**	0.12	470.37	0.5	68.3	33.73
**150**	0.12	470.37	0.6	80.5	59.71
**151**	0.12	470.37	0.7	94.4	88.54
**152**	0.12	470.37	0.8	100	100.00
**153**	0.12	470.37	0.9	100	100.00
**154**	0.12	498.77	0.1	57.8	1.89
**155**	0.12	498.77	0.2	56.4	3.14
**156**	0.12	498.77	0.3	57.8	8.76
**157**	0.12	498.77	0.4	59.7	14.51
**158**	0.12	498.77	0.5	70.9	39.17
**159**	0.12	498.77	0.6	82.9	64.67
**160**	0.12	498.77	0.7	98.3	96.52
**161**	0.12	498.77	0.8	100	100.00
**162**	0.12	498.77	0.9	100	100.00
**163**	0.12	527.16	0.1	58.6	3.75
**164**	0.12	527.16	0.2	56.6	3.59
**165**	0.12	527.16	0.3	56.8	6.59
**166**	0.12	527.16	0.4	61.3	17.90
**167**	0.12	527.16	0.5	73.2	43.97
**168**	0.12	527.16	0.6	84.5	67.98
**169**	0.12	527.16	0.7	99.9	99.80
**170**	0.12	527.16	0.8	100	100.00
**171**	0.12	527.16	0.9	100	100.00
**172**	0.12	555.56	0.1	58.2	2.82
**173**	0.12	555.56	0.2	56.8	4.03
**174**	0.12	555.56	0.3	58.5	10.27
**175**	0.12	555.56	0.4	62.5	20.45
**176**	0.12	555.56	0.5	74.9	47.53
**177**	0.12	555.56	0.6	88.5	76.24
**178**	0.12	555.56	0.7	100	100.00
**179**	0.12	555.56	0.8	100	100.00
**180**	0.12	555.56	0.9	100	100.00
**181**	0.23	300	0.1	57.4	0.96
**182**	0.23	300	0.2	55.2	0.48
**183**	0.23	300	0.3	54.8	2.27
**184**	0.23	300	0.4	54.5	3.48
**185**	0.23	300	0.5	55.3	6.55
**186**	0.23	300	0.6	57.7	12.61
**187**	0.23	300	0.7	58.3	14.70
**188**	0.23	300	0.8	67.7	34.49
**189**	0.23	300	0.9	74.8	49.27
**190**	0.23	328.40	0.1	57.4	0.96
**191**	0.23	328.40	0.2	55.3	0.70
**192**	0.23	328.40	0.3	54.9	2.48
**193**	0.23	328.40	0.4	54.8	4.12
**194**	0.23	328.40	0.5	56.9	9.90
**195**	0.23	328.40	0.6	58.2	13.64
**196**	0.23	328.40	0.7	63.3	24.93

**197**	0.23	328.40	0.8	71.2	41.59
**198**	0.23	328.40	0.9	78.8	57.32
**199**	0.23	356.79	0.1	57.4	0.96
**200**	0.23	356.79	0.2	56.2	2.70
**201**	0.23	356.79	0.3	55.9	4.65
**202**	0.23	356.79	0.4	55.3	5.18
**203**	0.23	356.79	0.5	56.2	8.44
**204**	0.23	356.79	0.6	59.7	16.74
**205**	0.23	356.79	0.7	68	34.54
**206**	0.23	356.79	0.8	75.5	50.31
**207**	0.23	356.79	0.9	81.8	63.36
**208**	0.23	385.19	0.1	57.8	1.89
**209**	0.23	385.19	0.2	56	2.25
**210**	0.23	385.19	0.3	55	2.70
**211**	0.23	385.19	0.4	55.7	6.02
**212**	0.23	385.19	0.5	57	10.11
**213**	0.23	385.19	0.6	62.8	23.15
**214**	0.23	385.19	0.7	69.7	38.02
**215**	0.23	385.19	0.8	78	55.38
**216**	0.23	385.19	0.9	88.9	77.65
**217**	0.23	413.58	0.1	57.5	1.19
**218**	0.23	413.58	0.2	55.8	1.81
**219**	0.23	413.58	0.3	55.5	3.78
**220**	0.23	413.58	0.4	56.2	7.08
**221**	0.23	413.58	0.5	56.9	9.90
**222**	0.23	413.58	0.6	64.9	27.49
**223**	0.23	413.58	0.7	71.9	42.52
**224**	0.23	413.58	0.8	80.6	60.65
**225**	0.23	413.58	0.9	94.8	89.53
**226**	0.23	441.98	0.1	57.5	1.19
**227**	0.23	441.98	0.2	56.2	2.70
**228**	0.23	441.98	0.3	55.9	4.65
**229**	0.23	441.98	0.4	55.9	6.45
**230**	0.23	441.98	0.5	59.3	14.92
**231**	0.23	441.98	0.6	67	31.82
**232**	0.23	441.98	0.7	73.6	46.00
**233**	0.23	441.98	0.8	86.5	72.62
**234**	0.23	441.98	0.9	97.8	95.57
**235**	0.23	470.37	0.1	57.7	1.65
**236**	0.23	470.37	0.2	56.2	2.70
**237**	0.23	470.37	0.3	55.3	3.35
**238**	0.23	470.37	0.4	56.7	8.15
**239**	0.23	470.37	0.5	62.3	21.19
**240**	0.23	470.37	0.6	69.5	36.99
**241**	0.23	470.37	0.7	76.6	52.13
**242**	0.23	470.37	0.8	91.4	82.56
**243**	0.23	470.37	0.9	99.5	98.99
**244**	0.23	498.77	0.1	57.7	1.65
**245**	0.23	498.77	0.2	56.4	3.14
**246**	0.23	498.77	0.3	55.9	4.65
**247**	0.23	498.77	0.4	56.1	6.87
**248**	0.23	498.77	0.5	63.2	23.07
**249**	0.23	498.77	0.6	70.3	38.64
**250**	0.23	498.77	0.7	79.6	58.27

**251**	0.23	498.77	0.8	94.9	89.66
**252**	0.23	498.77	0.9	99.5	98.99
**253**	0.23	527.16	0.1	57.5	1.19
**254**	0.23	527.16	0.2	56.2	2.70
**255**	0.23	527.16	0.3	56.5	5.94
**256**	0.23	527.16	0.4	57.5	9.84
**257**	0.23	527.16	0.5	65.2	27.25
**258**	0.23	527.16	0.6	71.9	41.95
**259**	0.23	527.16	0.7	84.2	67.68
**260**	0.23	527.16	0.8	97	93.92
**261**	0.23	527.16	0.9	99.9	99.80
**262**	0.23	555.56	0.1	57.5	1.19
**263**	0.23	555.56	0.2	56.6	3.59
**264**	0.23	555.56	0.3	56.4	5.73
**265**	0.23	555.56	0.4	58.9	12.81
**266**	0.23	555.56	0.5	66	28.92
**267**	0.23	555.56	0.6	73.8	45.87
**268**	0.23	555.56	0.7	87.1	73.61
**269**	0.23	555.56	0.8	99	97.97
**270**	0.23	555.56	0.9	99.8	99.60
**271**	0.34	300	0.1	57.3	0.72
**272**	0.34	300	0.2	55.5	1.14
**273**	0.34	300	0.3	54.6	1.84
**274**	0.34	300	0.4	54.8	4.12
**275**	0.34	300	0.5	55.1	6.14
**276**	0.34	300	0.6	55	7.03
**277**	0.34	300	0.7	57.3	12.65
**278**	0.34	300	0.8	63.2	25.36
**279**	0.34	300	0.9	67.6	34.77
**280**	0.34	328.40	0.1	57.1	0.26
**281**	0.34	328.40	0.2	56	2.25
**282**	0.34	328.40	0.3	55.1	2.92
**283**	0.34	328.40	0.4	54.6	3.69
**284**	0.34	328.40	0.5	54.9	5.72
**285**	0.34	328.40	0.6	55.7	8.48
**286**	0.34	328.40	0.7	59.6	17.36
**287**	0.34	328.40	0.8	65.8	30.63
**288**	0.34	328.40	0.9	70.1	39.80
**289**	0.34	356.79	0.1	57.9	2.12
**290**	0.34	356.79	0.2	55.8	1.81
**291**	0.34	356.79	0.3	54.4	1.40
**292**	0.34	356.79	0.4	54.8	4.12
**293**	0.34	356.79	0.5	55.4	6.76
**294**	0.34	356.79	0.6	57.6	12.40
**295**	0.34	356.79	0.7	62.6	23.49
**296**	0.34	356.79	0.8	68.2	35.50
**297**	0.34	356.79	0.9	72.1	43.83
**298**	0.34	385.19	0.1	57.8	1.89
**299**	0.34	385.19	0.2	55.8	1.81
**300**	0.34	385.19	0.3	54.4	1.40
**301**	0.34	385.19	0.4	54.8	4.12
**302**	0.34	385.19	0.5	55.5	6.97
**303**	0.34	385.19	0.6	59.2	15.71
**304**	0.34	385.19	0.7	64.5	27.38

**305**	0.34	385.19	0.8	70.5	40.17
**306**	0.34	385.19	0.9	77.2	54.10
**307**	0.34	413.58	0.1	57.8	1.89
**308**	0.34	413.58	0.2	55.7	1.59
**309**	0.34	413.58	0.3	55.2	3.13
**310**	0.34	413.58	0.4	55.1	4.75
**311**	0.34	413.58	0.5	56.4	8.85
**312**	0.34	413.58	0.6	61.5	20.46
**313**	0.34	413.58	0.7	66.9	32.29
**314**	0.34	413.58	0.8	72.7	44.63
**315**	0.34	413.58	0.9	81.4	62.55
**316**	0.34	441.98	0.1	57.8	1.89
**317**	0.34	441.98	0.2	56	2.25
**318**	0.34	441.98	0.3	54.7	2.05
**319**	0.34	441.98	0.4	55.7	6.02
**320**	0.34	441.98	0.5	56.1	8.23
**321**	0.34	441.98	0.6	63.2	23.97
**322**	0.34	441.98	0.7	66.8	32.09
**323**	0.34	441.98	0.8	76.1	51.52
**324**	0.34	441.98	0.9	85.6	71.01
**325**	0.34	470.37	0.1	58.1	2.58
**326**	0.34	470.37	0.2	56.7	3.81
**327**	0.34	470.37	0.3	55.6	4.00
**328**	0.34	470.37	0.4	55.1	4.75
**329**	0.34	470.37	0.5	59.3	14.92
**330**	0.34	470.37	0.6	64.7	27.07
**331**	0.34	470.37	0.7	69.8	38.22
**332**	0.34	470.37	0.8	79.8	59.03
**333**	0.34	470.37	0.9	88.7	77.25
**334**	0.34	498.77	0.1	57.5	1.19
**335**	0.34	498.77	0.2	56	2.25
**336**	0.34	498.77	0.3	55.1	2.92
**337**	0.34	498.77	0.4	55.5	5.60
**338**	0.34	498.77	0.5	60.3	17.01
**339**	0.34	498.77	0.6	65.9	29.55
**340**	0.34	498.77	0.7	71.9	42.52
**341**	0.34	498.77	0.8	81.5	62.48
**342**	0.34	498.77	0.9	91.8	83.49
**343**	0.34	527.16	0.1	57.9	2.12
**344**	0.34	527.16	0.2	55.9	2.03
**345**	0.34	527.16	0.3	55.6	4.00
**346**	0.34	527.16	0.4	56.7	8.15
**347**	0.34	527.16	0.5	61.4	19.31
**348**	0.34	527.16	0.6	66.6	31.00
**349**	0.34	527.16	0.7	74.7	48.25
**350**	0.34	527.16	0.8	83.9	67.34
**351**	0.34	527.16	0.9	93.2	86.31
**352**	0.34	555.56	0.1	58	2.35
**353**	0.34	555.56	0.2	55.6	1.36
**354**	0.34	555.56	0.3	55.7	4.21
**355**	0.34	555.56	0.4	56.5	7.72
**356**	0.34	555.56	0.5	61	18.47
**357**	0.34	555.56	0.6	67.9	33.68
**358**	0.34	555.56	0.7	76.7	52.34

**359**	0.34	555.56	0.8	85.5	70.59
**360**	0.34	555.56	0.9	94.7	89.33
**361**	0.45	300	0.1	58.3	3.05
**362**	0.45	300	0.2	55.1	0.25
**363**	0.45	300	0.3	54.6	1.84
**364**	0.45	300	0.4	54.1	2.63
**365**	0.45	300	0.5	53.7	3.21
**366**	0.45	300	0.6	53.9	4.76
**367**	0.45	300	0.7	54.1	6.11
**368**	0.45	300	0.8	58.9	16.64
**369**	0.45	300	0.9	63.1	25.71
**370**	0.45	328.40	0.1	57.6	1.42
**371**	0.45	328.40	0.2	55.7	1.59
**372**	0.45	328.40	0.3	54.4	1.40
**373**	0.45	328.40	0.4	54.3	3.05
**374**	0.45	328.40	0.5	54.7	5.30
**375**	0.45	328.40	0.6	54.9	6.83
**376**	0.45	328.40	0.7	57.6	13.27
**377**	0.45	328.40	0.8	61.1	21.10
**378**	0.45	328.40	0.9	64.5	28.53
**379**	0.45	356.79	0.1	58.3	3.05
**380**	0.45	356.79	0.2	55.3	0.70
**381**	0.45	356.79	0.3	54	0.54
**382**	0.45	356.79	0.4	54.4	3.27
**383**	0.45	356.79	0.5	54.5	4.88
**384**	0.45	356.79	0.6	55.1	7.24
**385**	0.45	356.79	0.7	59.8	17.77
**386**	0.45	356.79	0.8	63.6	26.17
**387**	0.45	356.79	0.9	66.7	32.96
**388**	0.45	385.19	0.1	57.7	1.65
**389**	0.45	385.19	0.2	55.1	0.25
**390**	0.45	385.19	0.3	54.3	1.19
**391**	0.45	385.19	0.4	54.8	4.12
**392**	0.45	385.19	0.5	55.2	6.34
**393**	0.45	385.19	0.6	57.4	11.99
**394**	0.45	385.19	0.7	62	22.27
**395**	0.45	385.19	0.8	66	31.04
**396**	0.45	385.19	0.9	71.3	42.22
**397**	0.45	413.58	0.1	57.5	1.19
**398**	0.45	413.58	0.2	55.6	1.36
**399**	0.45	413.58	0.3	55.5	3.78
**400**	0.45	413.58	0.4	54.5	3.48
**401**	0.45	413.58	0.5	55.3	6.55
**402**	0.45	413.58	0.6	59.3	15.92
**403**	0.45	413.58	0.7	62.6	23.49
**404**	0.45	413.58	0.8	67.4	33.88
**405**	0.45	413.58	0.9	74.2	48.06
**406**	0.45	441.98	0.1	57.6	1.42
**407**	0.45	441.98	0.2	55.7	1.59
**408**	0.45	441.98	0.3	55.3	3.35
**409**	0.45	441.98	0.4	55.1	4.75
**410**	0.45	441.98	0.5	55.8	7.60
**411**	0.45	441.98	0.6	60	17.36
**412**	0.45	441.98	0.7	64.4	27.18

**413**	0.45	441.98	0.8	70.5	40.17
**414**	0.45	441.98	0.9	77.6	54.90
**415**	0.45	470.37	0.1	57.7	1.65
**416**	0.45	470.37	0.2	55.6	1.36
**417**	0.45	470.37	0.3	55.3	3.35
**418**	0.45	470.37	0.4	55.3	5.18
**419**	0.45	470.37	0.5	57.3	10.73
**420**	0.45	470.37	0.6	60.8	19.01
**421**	0.45	470.37	0.7	65.6	29.63
**422**	0.45	470.37	0.8	73.4	46.05
**423**	0.45	470.37	0.9	80.2	60.14
**424**	0.45	498.77	0.1	58	2.35
**425**	0.45	498.77	0.2	55.4	0.92
**426**	0.45	498.77	0.3	55	2.70
**427**	0.45	498.77	0.4	54.9	4.33
**428**	0.45	498.77	0.5	58.5	13.24
**429**	0.45	498.77	0.6	61.9	21.29
**430**	0.45	498.77	0.7	68	34.54
**431**	0.45	498.77	0.8	75.6	50.51
**432**	0.45	498.77	0.9	81.4	62.55
**433**	0.45	527.16	0.1	57.5	1.19
**434**	0.45	527.16	0.2	56.2	2.70
**435**	0.45	527.16	0.3	55	2.70
**436**	0.45	527.16	0.4	55.6	5.81
**437**	0.45	527.16	0.5	59	14.29
**438**	0.45	527.16	0.6	63	23.56
**439**	0.45	527.16	0.7	70	38.63
**440**	0.45	527.16	0.8	76.8	52.94
**441**	0.45	527.16	0.9	83.1	65.98
**442**	0.45	555.56	0.1	57.5	1.19
**443**	0.45	555.56	0.2	56.2	2.70
**444**	0.45	555.56	0.3	55.3	3.35
**445**	0.45	555.56	0.4	55.8	6.24
**446**	0.45	555.56	0.5	59.6	15.54
**447**	0.45	555.56	0.6	64.7	27.07
**448**	0.45	555.56	0.7	71.4	41.50
**449**	0.45	555.56	0.8	77.9	55.17
**450**	0.45	555.56	0.9	85	69.80
**451**	0.56	300	0.1	57.6	1.42
**452**	0.56	300	0.2	55.5	1.14
**453**	0.56	300	0.3	54.6	1.84
**454**	0.56	300	0.4	54.2	2.84
**455**	0.56	300	0.5	53.8	3.42
**456**	0.56	300	0.6	54.7	6.41
**457**	0.56	300	0.7	54.4	6.72
**458**	0.56	300	0.8	58.1	15.02
**459**	0.56	300	0.9	60.5	20.48
**460**	0.56	328.40	0.1	57.5	1.19
**461**	0.56	328.40	0.2	55.8	1.81
**462**	0.56	328.40	0.3	54.6	1.84
**463**	0.56	328.40	0.4	53.7	1.78
**464**	0.56	328.40	0.5	53.7	3.21
**465**	0.56	328.40	0.6	54.1	5.17
**466**	0.56	328.40	0.7	56.1	10.20

**467**	0.56	328.40	0.8	58.9	16.64
**468**	0.56	328.40	0.9	61.2	21.89
**469**	0.56	356.79	0.1	58	2.35
**470**	0.56	356.79	0.2	55.7	1.59
**471**	0.56	356.79	0.3	54.3	1.19
**472**	0.56	356.79	0.4	54	2.42
**473**	0.56	356.79	0.5	54.8	5.51
**474**	0.56	356.79	0.6	55	7.03
**475**	0.56	356.79	0.7	58.1	14.29
**476**	0.56	356.79	0.8	60.9	20.69
**477**	0.56	356.79	0.9	63.8	27.12
**478**	0.56	385.19	0.1	57.7	1.65
**479**	0.56	385.19	0.2	55.9	2.03
**480**	0.56	385.19	0.3	55.2	3.13
**481**	0.56	385.19	0.4	54.5	3.48
**482**	0.56	385.19	0.5	54.8	5.51
**483**	0.56	385.19	0.6	56.3	9.72
**484**	0.56	385.19	0.7	58.8	15.72
**485**	0.56	385.19	0.8	62.3	23.53
**486**	0.56	385.19	0.9	67.2	33.97
**487**	0.56	413.58	0.1	57.2	0.49
**488**	0.56	413.58	0.2	55.6	1.36
**489**	0.56	413.58	0.3	55.1	2.92
**490**	0.56	413.58	0.4	54.4	3.27
**491**	0.56	413.58	0.5	55	5.93
**492**	0.56	413.58	0.6	58.3	13.85
**493**	0.56	413.58	0.7	60.4	18.99
**494**	0.56	413.58	0.8	64.5	28.00
**495**	0.56	413.58	0.9	69.5	38.60
**496**	0.56	441.98	0.1	57.4	0.96
**497**	0.56	441.98	0.2	56.2	2.70
**498**	0.56	441.98	0.3	54.9	2.48
**499**	0.56	441.98	0.4	54.9	4.33
**500**	0.56	441.98	0.5	55.6	7.18
**501**	0.56	441.98	0.6	57.8	12.82
**502**	0.56	441.98	0.7	61.4	21.04
**503**	0.56	441.98	0.8	67.2	33.47
**504**	0.56	441.98	0.9	73	45.64
**505**	0.56	470.37	0.1	57.6	1.42
**506**	0.56	470.37	0.2	55.7	1.59
**507**	0.56	470.37	0.3	55.4	3.57
**508**	0.56	470.37	0.4	54.7	3.90
**509**	0.56	470.37	0.5	56.1	8.23
**510**	0.56	470.37	0.6	59.2	15.71
**511**	0.56	470.37	0.7	63.1	24.52
**512**	0.56	470.37	0.8	69.9	38.95
**513**	0.56	470.37	0.9	74	47.66
**514**	0.56	498.77	0.1	57.9	2.12
**515**	0.56	498.77	0.2	56	2.25
**516**	0.56	498.77	0.3	54.9	2.48
**517**	0.56	498.77	0.4	54.1	2.63
**518**	0.56	498.77	0.5	57.3	10.73
**519**	0.56	498.77	0.6	60.7	18.81
**520**	0.56	498.77	0.7	65.3	29.02

**521**	0.56	498.77	0.8	71.2	41.59
**522**	0.56	498.77	0.9	75.5	50.68
**523**	0.56	527.16	0.1	57.7	1.65
**524**	0.56	527.16	0.2	55.9	2.03
**525**	0.56	527.16	0.3	55.7	4.21
**526**	0.56	527.16	0.4	55.2	4.96
**527**	0.56	527.16	0.5	58.8	13.87
**528**	0.56	527.16	0.6	61	19.43
**529**	0.56	527.16	0.7	67.1	32.70
**530**	0.56	527.16	0.8	72.8	44.83
**531**	0.56	527.16	0.9	78.1	55.91
**532**	0.56	555.56	0.1	57.5	1.19
**533**	0.56	555.56	0.2	55.4	0.92
**534**	0.56	555.56	0.3	54.4	1.40
**535**	0.56	555.56	0.4	55.4	5.39
**536**	0.56	555.56	0.5	57.8	11.78
**537**	0.56	555.56	0.6	61.7	20.87
**538**	0.56	555.56	0.7	69.1	36.79
**539**	0.56	555.56	0.8	73.2	45.64
**540**	0.56	555.56	0.9	77.6	54.90
**541**	0.67	300	0.1	57.6	1.42
**542**	0.67	300	0.2	55.9	2.03
**543**	0.67	300	0.3	54.1	0.76
**544**	0.67	300	0.4	53.7	1.78
**545**	0.67	300	0.5	53.3	2.37
**546**	0.67	300	0.6	53.2	3.31
**547**	0.67	300	0.7	54.1	6.11
**548**	0.67	300	0.8	56.7	12.18
**549**	0.67	300	0.9	58.9	17.26
**550**	0.67	328.40	0.1	57.4	0.96
**551**	0.67	328.40	0.2	55.2	0.48
**552**	0.67	328.40	0.3	54.6	1.84
**553**	0.67	328.40	0.4	53.9	2.21
**554**	0.67	328.40	0.5	54	3.84
**555**	0.67	328.40	0.6	53.7	4.35
**556**	0.67	328.40	0.7	54.8	7.54
**557**	0.67	328.40	0.8	58.1	15.02
**558**	0.67	328.40	0.9	59.7	18.87
**559**	0.67	356.79	0.1	57.9	2.12
**560**	0.67	356.79	0.2	56	2.25
**561**	0.67	356.79	0.3	54.4	1.40
**562**	0.67	356.79	0.4	54.6	3.69
**563**	0.67	356.79	0.5	53.6	3.00
**564**	0.67	356.79	0.6	54.3	5.59
**565**	0.67	356.79	0.7	57.1	12.24
**566**	0.67	356.79	0.8	59.3	17.45
**567**	0.67	356.79	0.9	61.9	23.30
**568**	0.67	385.19	0.1	57.8	1.89
**569**	0.67	385.19	0.2	55.3	0.70
**570**	0.67	385.19	0.3	55.2	3.13
**571**	0.67	385.19	0.4	54.6	3.69
**572**	0.67	385.19	0.5	54.1	4.04
**573**	0.67	385.19	0.6	55.4	7.86
**574**	0.67	385.19	0.7	57.5	13.06

**575**	0.67	385.19	0.8	60.5	19.88
**576**	0.67	385.19	0.9	64.2	27.93
**577**	0.67	413.58	0.1	57.4	0.96
**578**	0.67	413.58	0.2	56.2	2.70
**579**	0.67	413.58	0.3	54.9	2.48
**580**	0.67	413.58	0.4	54.8	4.12
**581**	0.67	413.58	0.5	54.7	5.30
**582**	0.67	413.58	0.6	56.5	10.13
**583**	0.67	413.58	0.7	59.2	16.54
**584**	0.67	413.58	0.8	61.8	22.52
**585**	0.67	413.58	0.9	67.2	33.97
**586**	0.67	441.98	0.1	58	2.35
**587**	0.67	441.98	0.2	55.9	2.03
**588**	0.67	441.98	0.3	54.9	2.48
**589**	0.67	441.98	0.4	54.6	3.69
**590**	0.67	441.98	0.5	54.8	5.51
**591**	0.67	441.98	0.6	56.9	10.96
**592**	0.67	441.98	0.7	59.6	17.36
**593**	0.67	441.98	0.8	65.1	29.21
**594**	0.67	441.98	0.9	68.8	37.19
**595**	0.67	470.37	0.1	58.2	2.82
**596**	0.67	470.37	0.2	56.1	2.48
**597**	0.67	470.37	0.3	55	2.70
**598**	0.67	470.37	0.4	54.6	3.69
**599**	0.67	470.37	0.5	55	5.93
**600**	0.67	470.37	0.6	58	13.23
**601**	0.67	470.37	0.7	61.4	21.04
**602**	0.67	470.37	0.8	66.7	32.46
**603**	0.67	470.37	0.9	71.7	43.03
**604**	0.67	498.77	0.1	57.6	1.42
**605**	0.67	498.77	0.2	55.9	2.03
**606**	0.67	498.77	0.3	54.7	2.05
**607**	0.67	498.77	0.4	54.3	3.05
**608**	0.67	498.77	0.5	56.5	9.06
**609**	0.67	498.77	0.6	59	15.30
**610**	0.67	498.77	0.7	63.3	24.93
**611**	0.67	498.77	0.8	68.1	35.30
**612**	0.67	498.77	0.9	71.3	42.22
**613**	0.67	527.16	0.1	58	2.35
**614**	0.67	527.16	0.2	55.8	1.81
**615**	0.67	527.16	0.3	54.6	1.84
**616**	0.67	527.16	0.4	55	4.54
**617**	0.67	527.16	0.5	57.1	10.32
**618**	0.67	527.16	0.6	59.7	16.74
**619**	0.67	527.16	0.7	63.9	26.15
**620**	0.67	527.16	0.8	69.5	38.14
**621**	0.67	527.16	0.9	72.3	44.23
**622**	0.67	555.56	0.1	57.2	0.49
**623**	0.67	555.56	0.2	55.4	0.92
**624**	0.67	555.56	0.3	54.9	2.48
**625**	0.67	555.56	0.4	55	4.54
**626**	0.67	555.56	0.5	57.5	11.15
**627**	0.67	555.56	0.6	60.8	19.01
**628**	0.67	555.56	0.7	66.2	30.86

**629**	0.67	555.56	0.8	70.5	40.17
**630**	0.67	555.56	0.9	73.5	46.65
**631**	0.78	300	0.1	57.7	1.65
**632**	0.78	300	0.2	55.6	1.37
**633**	0.78	300	0.3	54	0.54
**634**	0.78	300	0.4	54	2.42
**635**	0.78	300	0.5	53.2	2.16
**636**	0.78	300	0.6	53.1	3.11
**637**	0.78	300	0.7	52.9	3.65
**638**	0.78	300	0.8	54.8	8.32
**639**	0.78	300	0.9	57	13.43
**640**	0.78	328.40	0.1	57.9	2.12
**641**	0.78	328.40	0.2	56.3	2.92
**642**	0.78	328.40	0.3	54	0.54
**643**	0.78	328.40	0.4	53.7	1.78
**644**	0.78	328.40	0.5	53.8	3.42
**645**	0.78	328.40	0.6	53.6	4.14
**646**	0.78	328.40	0.7	54.5	6.93
**647**	0.78	328.40	0.8	56.8	12.38
**648**	0.78	328.40	0.9	57.4	14.24
**649**	0.78	356.79	0.1	57.7	1.65
**650**	0.78	356.79	0.2	55.8	1.81
**651**	0.78	356.79	0.3	53.8	0.11
**652**	0.78	356.79	0.4	54.1	2.63
**653**	0.78	356.79	0.5	53.3	2.37
**654**	0.78	356.79	0.6	53.7	4.35
**655**	0.78	356.79	0.7	56.3	10.61
**656**	0.78	356.79	0.8	58.1	15.02
**657**	0.78	356.79	0.9	59.1	17.66
**658**	0.78	385.19	0.1	58	2.35
**659**	0.78	385.19	0.2	55.6	1.36
**660**	0.78	385.19	0.3	54.9	2.48
**661**	0.78	385.19	0.4	54.5	3.48
**662**	0.78	385.19	0.5	53.8	3.42
**663**	0.78	385.19	0.6	55.4	7.86
**664**	0.78	385.19	0.7	57.3	12.65
**665**	0.78	385.19	0.8	59.7	18.26
**666**	0.78	385.19	0.9	62.2	23.90
**667**	0.78	413.58	0.1	57.9	2.12
**668**	0.78	413.58	0.2	55.6	1.36
**669**	0.78	413.58	0.3	54.9	2.48
**670**	0.78	413.58	0.4	54.5	3.48
**671**	0.78	413.58	0.5	53.9	3.63
**672**	0.78	413.58	0.6	55.5	8.07
**673**	0.78	413.58	0.7	58.2	14.49
**674**	0.78	413.58	0.8	60.5	19.88
**675**	0.78	413.58	0.9	64.5	28.53
**676**	0.78	441.98	0.1	58	2.35
**677**	0.78	441.98	0.2	56	2.25
**678**	0.78	441.98	0.3	54.6	1.84
**679**	0.78	441.98	0.4	54.7	3.90
**680**	0.78	441.98	0.5	54.2	4.25
**681**	0.78	441.98	0.6	56.8	10.75
**682**	0.78	441.98	0.7	59.1	16.34

**683**	0.78	441.98	0.8	62.5	23.94
**684**	0.78	441.98	0.9	67.4	34.37
**685**	0.78	470.37	0.1	57.8	1.89
**686**	0.78	470.37	0.2	55.6	1.36
**687**	0.78	470.37	0.3	55	2.70
**688**	0.78	470.37	0.4	54.6	3.69
**689**	0.78	470.37	0.5	55.4	6.76
**690**	0.78	470.37	0.6	56.8	10.75
**691**	0.78	470.37	0.7	59.3	16.74
**692**	0.78	470.37	0.8	63.7	26.37
**693**	0.78	470.37	0.9	68.9	37.39
**694**	0.78	498.77	0.1	57.9	2.12
**695**	0.78	498.77	0.2	55.7	1.59
**696**	0.78	498.77	0.3	55.4	3.57
**697**	0.78	498.77	0.4	54	2.42
**698**	0.78	498.77	0.5	55.9	7.81
**699**	0.78	498.77	0.6	57.6	12.40
**700**	0.78	498.77	0.7	61.1	20.43
**701**	0.78	498.77	0.8	66.3	31.65
**702**	0.78	498.77	0.9	69.9	39.40
**703**	0.78	527.16	0.1	57.9	2.12
**704**	0.78	527.16	0.2	56.3	2.92
**705**	0.78	527.16	0.3	55	2.70
**706**	0.78	527.16	0.4	55.1	4.75
**707**	0.78	527.16	0.5	56.2	8.43
**708**	0.78	527.16	0.6	58	13.23
**709**	0.78	527.16	0.7	62.5	23.29
**710**	0.78	527.16	0.8	67.2	33.47
**711**	0.78	527.16	0.9	69.2	37.99
**712**	0.78	555.56	0.1	57.6	1.42
**713**	0.78	555.56	0.2	55.9	2.03
**714**	0.78	555.56	0.3	54.5	1.62
**715**	0.78	555.56	0.4	54.8	4.11
**716**	0.78	555.56	0.5	57.2	10.53
**717**	0.78	555.56	0.6	59.4	16.12
**718**	0.78	555.56	0.7	64	26.36
**719**	0.78	555.56	0.8	67.7	34.49
**720**	0.78	555.56	0.9	71.2	42.02
**721**	0.89	300	0.1	57.6	1.42
**722**	0.89	300	0.2	55.6	1.37
**723**	0.89	300	0.3	54.3	1.19
**724**	0.89	300	0.4	53.9	2.21
**725**	0.89	300	0.5	53.6	3.00
**726**	0.89	300	0.6	53	2.90
**727**	0.89	300	0.7	53.2	4.27
**728**	0.89	300	0.8	54.8	8.32
**729**	0.89	300	0.9	55.7	10.81
**730**	0.89	328.40	0.1	58	2.35
**731**	0.89	328.40	0.2	55.4	0.92
**732**	0.89	328.40	0.3	54.3	1.19
**733**	0.89	328.40	0.4	53.8	1.99
**734**	0.89	328.40	0.5	53.1	1.95
**735**	0.89	328.40	0.6	52.9	2.69
**736**	0.89	328.40	0.7	53.3	4.47

**737**	0.89	328.40	0.8	56	10.76
**738**	0.89	328.40	0.9	57.2	13.83
**739**	0.89	356.79	0.1	56.9	-0.21
**740**	0.89	356.79	0.2	55.3	0.70
**741**	0.89	356.79	0.3	54.8	2.27
**742**	0.89	356.79	0.4	53.5	1.36
**743**	0.89	356.79	0.5	54.5	4.88
**744**	0.89	356.79	0.6	54.2	5.38
**745**	0.89	356.79	0.7	56	9.99
**746**	0.89	356.79	0.8	56.8	12.38
**747**	0.89	356.79	0.9	58.1	15.65
**748**	0.89	385.19	0.1	57.8	1.89
**749**	0.89	385.19	0.2	55.6	1.36
**750**	0.89	385.19	0.3	54.7	2.05
**751**	0.89	385.19	0.4	54	2.42
**752**	0.89	385.19	0.5	53.4	2.58
**753**	0.89	385.19	0.6	54.5	6.00
**754**	0.89	385.19	0.7	56.1	10.20
**755**	0.89	385.19	0.8	57.4	13.60
**756**	0.89	385.19	0.9	61.5	22.49
**757**	0.89	413.58	0.1	57.9	2.12
**758**	0.89	413.58	0.2	55.9	2.03
**759**	0.89	413.58	0.3	54.8	2.27
**760**	0.89	413.58	0.4	53.5	1.36
**761**	0.89	413.58	0.5	53	1.75
**762**	0.89	413.58	0.6	55.2	7.45
**763**	0.89	413.58	0.7	57.3	12.65
**764**	0.89	413.58	0.8	60	18.87
**765**	0.89	413.58	0.9	61.4	22.29
**766**	0.89	441.98	0.1	57.3	0.72
**767**	0.89	441.98	0.2	56.2	2.70
**768**	0.89	441.98	0.3	54.2	0.97
**769**	0.89	441.98	0.4	53.6	1.57
**770**	0.89	441.98	0.5	54.2	4.25
**771**	0.89	441.98	0.6	56.1	9.30
**772**	0.89	441.98	0.7	56.6	11.22
**773**	0.89	441.98	0.8	60.5	19.88
**774**	0.89	441.98	0.9	64.7	28.93
**775**	0.89	470.37	0.1	57.5	1.19
**776**	0.89	470.37	0.2	55.7	1.59
**777**	0.89	470.37	0.3	55	2.70
**778**	0.89	470.37	0.4	54.2	2.84
**779**	0.89	470.37	0.5	54.9	5.72
**780**	0.89	470.37	0.6	56.4	9.92
**781**	0.89	470.37	0.7	58.4	14.90
**782**	0.89	470.37	0.8	62.9	24.75
**783**	0.89	470.37	0.9	66.9	33.36
**784**	0.89	498.77	0.1	57.9	2.12
**785**	0.89	498.77	0.2	55.2	0.48
**786**	0.89	498.77	0.3	54.8	2.27
**787**	0.89	498.77	0.4	53.8	1.99
**788**	0.89	498.77	0.5	55	5.93
**789**	0.89	498.77	0.6	57.3	11.78
**790**	0.89	498.77	0.7	60.3	18.79

**791**	0.89	498.77	0.8	64.1	27.18
**792**	0.89	498.77	0.9	66.7	32.96
**793**	0.89	527.16	0.1	57.7	1.65
**794**	0.89	527.16	0.2	56.1	2.48
**795**	0.89	527.16	0.3	54.8	2.27
**796**	0.89	527.16	0.4	54.5	3.48
**797**	0.89	527.16	0.5	55.9	7.81
**798**	0.89	527.16	0.6	57.1	11.37
**799**	0.89	527.16	0.7	61.3	20.84
**800**	0.89	527.16	0.8	64.4	27.79
**801**	0.89	527.16	0.9	67.4	34.37
**802**	0.89	555.56	0.1	57.6	1.42
**803**	0.89	555.56	0.2	55.5	1.14
**804**	0.89	555.56	0.3	54.9	2.48
**805**	0.89	555.56	0.4	54.9	4.33
**806**	0.89	555.56	0.5	56	8.02
**807**	0.89	555.56	0.6	58.3	13.85
**808**	0.89	555.56	0.7	62.8	23.90
**809**	0.89	555.56	0.8	65.5	30.02
**810**	0.89	555.56	0.9	68.5	36.58
**811**	1	300	0.1	58	2.35
**812**	1	300	0.2	55.1	0.25
**813**	1	300	0.3	54.2	0.97
**814**	1	300	0.4	53.7	1.78
**815**	1	300	0.5	53.1	1.95
**816**	1	300	0.6	53	2.90
**817**	1	300	0.7	52.9	3.65
**818**	1	300	0.8	53.2	5.08
**819**	1	300	0.9	55.6	10.61
**820**	1	328.40	0.1	57.4	0.96
**821**	1	328.40	0.2	56	2.25
**822**	1	328.40	0.3	54.7	2.05
**823**	1	328.40	0.4	54	2.42
**824**	1	328.40	0.5	53.4	2.58
**825**	1	328.40	0.6	52.6	2.07
**826**	1	328.40	0.7	53.8	5.49
**827**	1	328.40	0.8	56.1	10.96
**828**	1	328.40	0.9	56.1	11.62
**829**	1	356.79	0.1	58.1	2.58
**830**	1	356.79	0.2	55.4	0.92
**831**	1	356.79	0.3	54.2	0.97
**832**	1	356.79	0.4	53.6	1.57
**833**	1	356.79	0.5	53.6	3.00
**834**	1	356.79	0.6	53.8	4.55
**835**	1	356.79	0.7	55.5	8.97
**836**	1	356.79	0.8	56.3	11.36
**837**	1	356.79	0.9	56.3	12.02
**838**	1	385.19	0.1	57.5	1.19
**839**	1	385.19	0.2	55.8	1.81
**840**	1	385.19	0.3	54.1	0.76
**841**	1	385.19	0.4	53.4	1.15
**842**	1	385.19	0.5	53.6	3.00
**843**	1	385.19	0.6	54.7	6.41
**844**	1	385.19	0.7	55.3	8.56

**845**	1	385.19	0.8	56.7	12.18
**846**	1	385.19	0.9	59.7	18.87
**847**	1	413.58	0.1	57.6	1.42
**848**	1	413.58	0.2	55.7	1.59
**849**	1	413.58	0.3	54.5	1.62
**850**	1	413.58	0.4	53.7	1.78
**851**	1	413.58	0.5	53.6	3.00
**852**	1	413.58	0.6	55.3	7.65
**853**	1	413.58	0.7	56.1	10.20
**854**	1	413.58	0.8	58.1	15.02
**855**	1	413.58	0.9	60.9	21.28
**856**	1	441.98	0.1	57.5	1.19
**857**	1	441.98	0.2	55.4	0.92
**858**	1	441.98	0.3	54.7	2.05
**859**	1	441.98	0.4	54.1	2.63
**860**	1	441.98	0.5	54.2	4.25
**861**	1	441.98	0.6	55.4	7.86
**862**	1	441.98	0.7	57.2	12.45
**863**	1	441.98	0.8	60.1	19.07
**864**	1	441.98	0.9	62.7	24.91
**865**	1	470.37	0.1	57.5	1.19
**866**	1	470.37	0.2	55.9	2.03
**867**	1	470.37	0.3	54.8	2.27
**868**	1	470.37	0.4	54	2.42
**869**	1	470.37	0.5	54.5	4.88
**870**	1	470.37	0.6	56	9.10
**871**	1	470.37	0.7	57.7	13.47
**872**	1	470.37	0.8	60.7	20.29
**873**	1	470.37	0.9	63.9	27.32
**874**	1	498.77	0.1	58	2.35
**875**	1	498.77	0.2	56.1	2.48
**876**	1	498.77	0.3	54.8	2.27
**877**	1	498.77	0.4	53.8	1.99
**878**	1	498.77	0.5	54.8	5.51
**879**	1	498.77	0.6	56.4	9.92
**880**	1	498.77	0.7	59.2	16.54
**881**	1	498.77	0.8	62.1	23.13
**882**	1	498.77	0.9	65.4	30.34
**883**	1	527.16	0.1	58.1	2.58
**884**	1	527.16	0.2	55.6	1.36
**885**	1	527.16	0.3	54.5	1.62
**886**	1	527.16	0.4	54.1	2.63
**887**	1	527.16	0.5	56.1	8.23
**888**	1	527.16	0.6	57.1	11.37
**889**	1	527.16	0.7	60.3	18.79
**890**	1	527.16	0.8	62.5	23.94
**891**	1	527.16	0.9	66.2	31.95
**892**	1	555.56	0.1	57.6	1.42
**893**	1	555.56	0.2	55.6	1.36
**894**	1	555.56	0.3	54.9	2.48
**895**	1	555.56	0.4	54.9	4.33
**896**	1	555.56	0.5	55.6	7.18
**897**	1	555.56	0.6	57.6	12.40
**898**	1	555.56	0.7	61.1	20.43

**899**	1	555.56	0.8	64.4	27.79
**900**	1	555.56	0.9	67.4	34.37
